# GANomics: bridging legacy and modern transcriptomic platforms for clinical applications

**DOI:** 10.1038/s41525-026-00585-w

**Published:** 2026-06-19

**Authors:** Leihong Wu, Hadi Salman, Weida Tong

**Affiliations:** 1 Division of Bioinformatics and Biostatistics, National Center for Toxicological Research, FDA, Jefferson, AR USA; 2 Department of Statistics, Texas A&M University, College Station, TX USA

**Keywords:** Cancer, Computational biology and bioinformatics

## Abstract

The evolution of transcriptomic technologies requires effective translation between legacy and modern platforms to fully leverage historical data. We present GANomics, a generative adversarial network (GAN) framework that enables bidirectional translation between microarray and RNA-seq data using a small number of samples profiled by both technologies. By integrating paired and unpaired samples through a pair-aware feedback loss, GANomics enforces one-to-one transcript mappings while preserving global gene expression distributions. Applied to a neuroblastoma cohort (*n* = 498; 10,042 genes) and benchmarked against six alternative methods, GANomics achieved high per-sample correlations ( > 0.96) between real and synthetic data with as few as ten paired profiles. With fifty paired profiles, it accurately recapitulated differential expression, maintained pathway-level rankings, and enabled a cross-platform classifier to transfer comparable to real data. Consistent performance across five additional benchmark datasets confirmed its robustness. By bridging legacy and contemporary transcriptomic data while retaining biological consistency, GANomics facilitates scalable data integration for biomarker reuse and the expansion of transcriptomic resources in clinical applications.

## Introduction

High-throughput transcriptomic profiling has transformed biomedical research over the past three decades^1–3^, enabling molecular insights into disease mechanisms^4,5^, biomarker discovery, and therapeutic development^6–8^. Large public repositories such as the Gene Expression Omnibus (GEO)^9^ continue to expand, offering a wealth of expression data that facilitates diagnostic and prognostic studies^10^. Yet, the field has undergone a major technological transition: microarrays, once the dominant platform, are being rapidly replaced by RNA sequencing (RNA-seq)^3,11^. This technological advancement is inevitable and will continue, thus raising the question of whether the data and applications from older technologies can be used alongside data generated with new ones.

While both platforms provide genome-wide expression measurements, their technical differences in measurement scale, distribution, and feature coverage pose a challenge for direct integration. Community efforts such as the FDA-led SEQC (Sequencing Quality Control) consortium demonstrate that biological interpretation that is broadly consistent across technologies, highlighting the value of legacy microarray data in the RNA-seq era^11–14^. However, effective reuse of this legacy data requires cross-platform translation. Forward translation (microarray → RNA-seq) allows old datasets to be integrated with more recent patient groups analyzed by RNA-seq, while reverse translation (RNA-seq → microarray) supports continuing application of established biomarkers and diagnostic signatures in the clinical setting^15,16^.

A variety of cross-platform normalization and integration strategies have been proposed, including training distribution matching (TDM)^17^, quantile normalization (QN)^18^, ComBat^19^, YuGene^20^, and CuBlock^21^. While these approaches can reduce platform biases, they typically operate at the dataset level rather than producing accurate sample-wise translations. Consequently, they may compress dynamic range, distort gene–gene relationships, or require dataset-specific tuning. Moreover, most evaluations focus on correlation or error metrics, offering limited evidence of preserved biological fidelity—such as differential expression, pathway enrichment, or predictive model performance—after translation. These limitations highlight the need for a generative framework that supports bidirectional, sample-wise translation while retaining biologically meaningful signal.

Recent advances in generative artificial intelligence (AI) have demonstrated remarkable success in cross-domain translation, from text-to-image models to adversarial frameworks in biomedical research^22–24^. Within transcriptomics, generative approaches have been applied to tasks such as virtual animal studies^25,26^, cross-organ translation^27,28^, and landmark-to-RNA-seq mapping^29^, exhibiting their potential for transcriptomic translation between microarray and RNA-seq. Most existing approaches are limited to one-way translation, rely on additional network modules, or fail to fully utilize paired samples when available. To address these limitations, we developed GANomics, a pair-aware generative framework that jointly integrates paired and unpaired data within a single training process. By introducing a feedback loss on paired profiles alongside adversarial and cycle-consistency objectives, GANomics achieves sample-wise, bidirectional translation between microarray and RNA-seq platforms while preserving biological consistency with only a small number of samples profiled by both technologies.

Utilizing a Neuroblastoma (NB) dataset, which includes 498 samples profiled by both microarrays and RNAseq, we evaluated the GANomics’s ability to generate high-quality synthetic profiles, assessed how many samples with paired data are needed for reliable translation, and examined whether the synthetic profiles support downstream predictive modeling and biological interpretation such as (1) Do synthetic profiles retain biological mechanisms through differential expression analysis and pathway enrichment? (2) Can synthetic profiles be used to develop a reliable predictive classifier for diagnosis and prognosis? and (3) Can a clinical sample with synthetic data be accurately diagnosed by a classifier developed on real data? In addition, we also benchmarked GANomics against six alternative methods and five additional clinical datasets. We demonstrated the potential utility of GANomics as a scalable and practical solution for integrating legacy and modern datasets to support transcriptomic innovation.

## Results

### GANomics overview and training performance

**Fig. 1:**
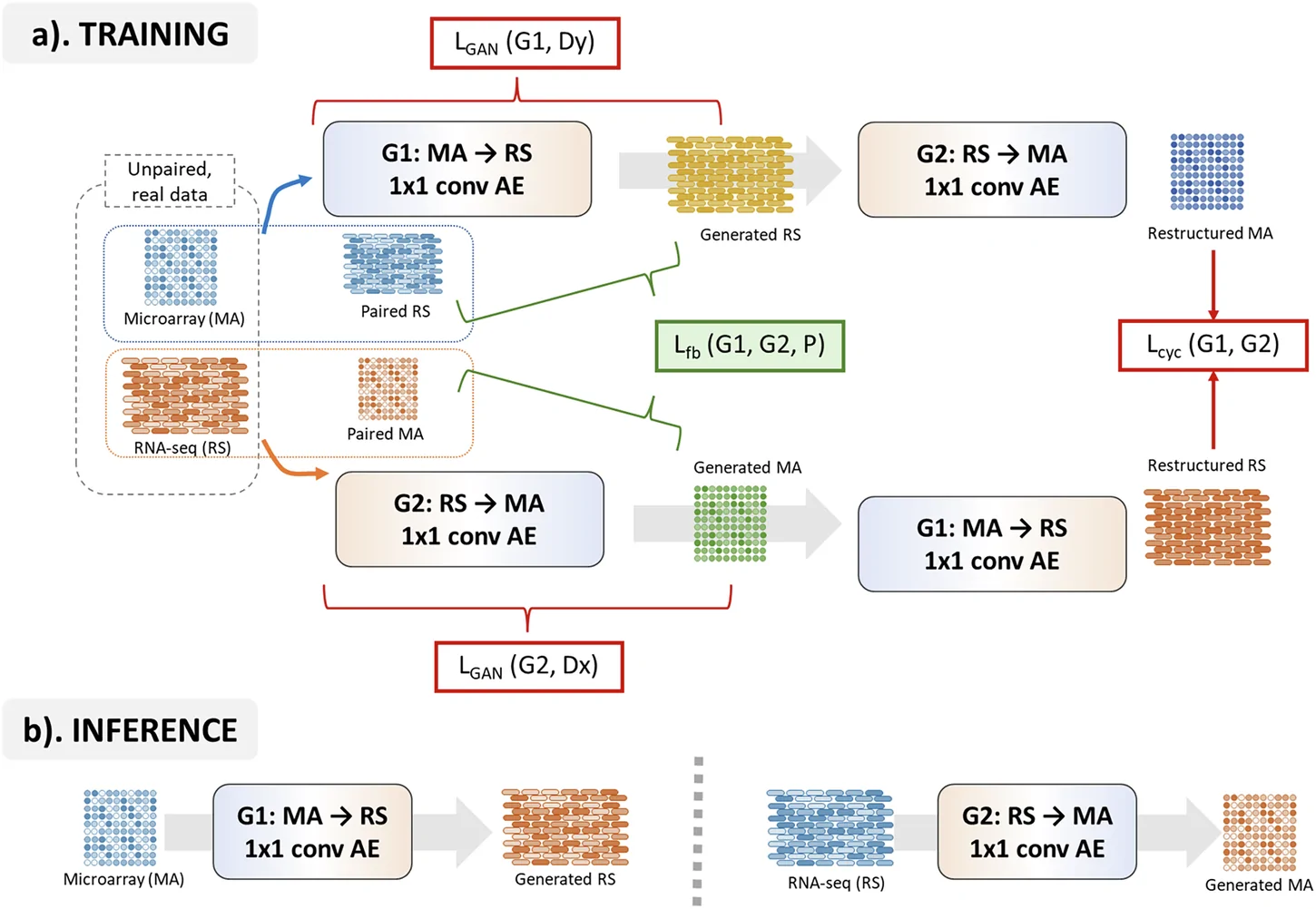
Overall GANomics framework. **a** GANomics training scheme. Similar to CycleGAN, a set of unpaired microarray (MA) and RNA-seq (RS) profiles was used for training the generator and discriminator networks. In addition, paired samples were used to measure a newly introduced feedback loss (fb), along with the generator loss (G), discriminator loss (D), and cycle loss (cyc). This cycle-consistent approach ensures accurate cross-platform translation while preserving biological fidelity. **b** During the inference stage, the trained generators require only a single input to produce a synthetic profile that mimics the desired target distribution.

GANomics utilizes both paired and unpaired data within the same training step. In each iteration, we input two microarray–RNAseq pairs, a paired set from the same biological sample, and an unpaired set formed by independently shuffling samples across platforms. The paired set anchors a feedback loss that drives G_MA→RS_ and G_RS→MA_ to reproduce the sample-specific counterpart, while the unpaired set supports adversarial and cycle-consistency objectives that align platform-level distributions without one-to-one correspondence. By optimizing on both pairs in every loop, GANomics simultaneously learns profile-wise mappings and platform-wise structure, enabling accurate cross-platform translation (Fig. 1).

**Fig. 2:**
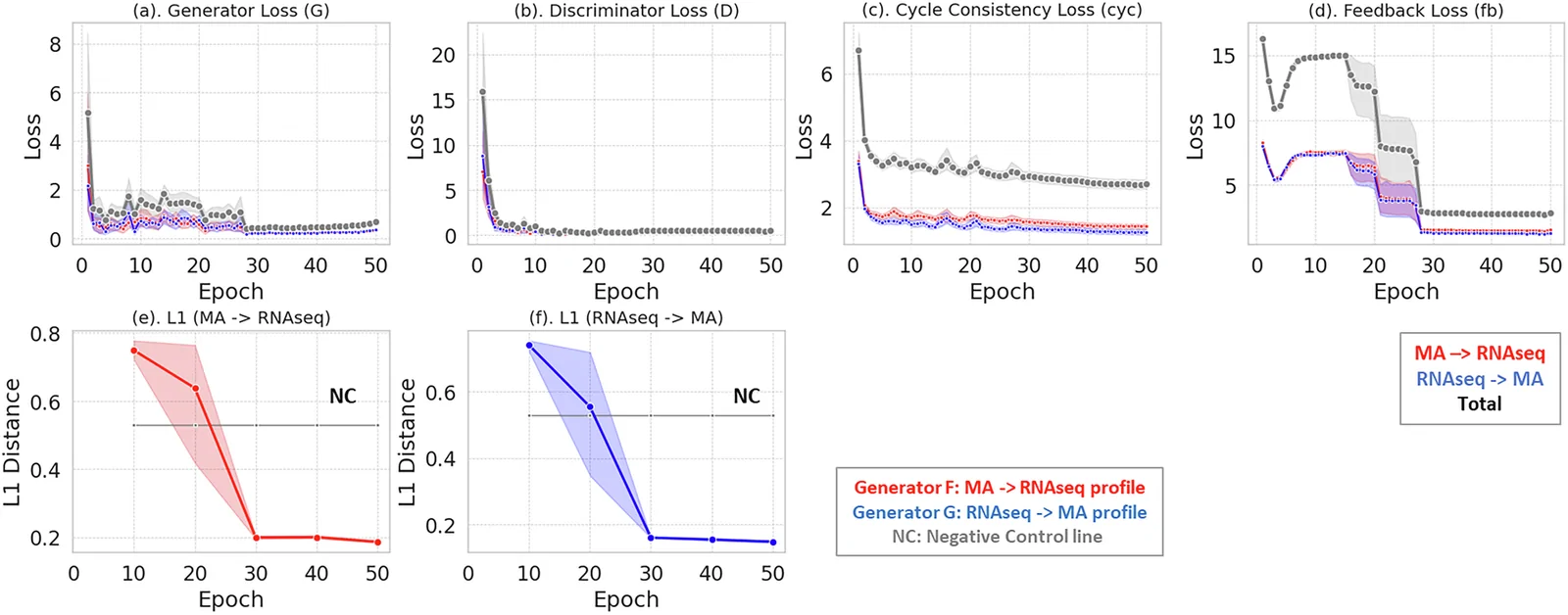
GANomics training performance. **a** overall Generator losses (G1 and G2); **b** overall discriminator losses; **c** overall cycle consistency losses (cyc), and **d** overall feedback losses during the training process. Losses converged after 30 epochs. **e**, **f** Averaged l1-distance between synthetic and real **e** RNA-seq and **f** microarray profiles. The negative control (NC) line represents the l1 distance between the microarray and RNA-seq profiles of the same testing sample.

We first evaluated GANomics on the NB dataset (*n* = 498). Using 398 paired profiles for training and holding out 100 for testing, we repeated the train/test split five times for robustness. Training converged in ~30 epochs, with generator, discriminator, cycle-consistency, and feedback losses stabilizing concordantly (Fig. 2a–d). The per-sample L1 distance between real microarray and RNA-seq profiles from the same sample was used as a baseline; translations with lower distances were deemed successful. GANomics fell well below the baseline on the 100 held-out samples (Fig. 2e, f).

### Hyperparameter sensitivity analysis of *β* and *λ*

**Fig. 3:**
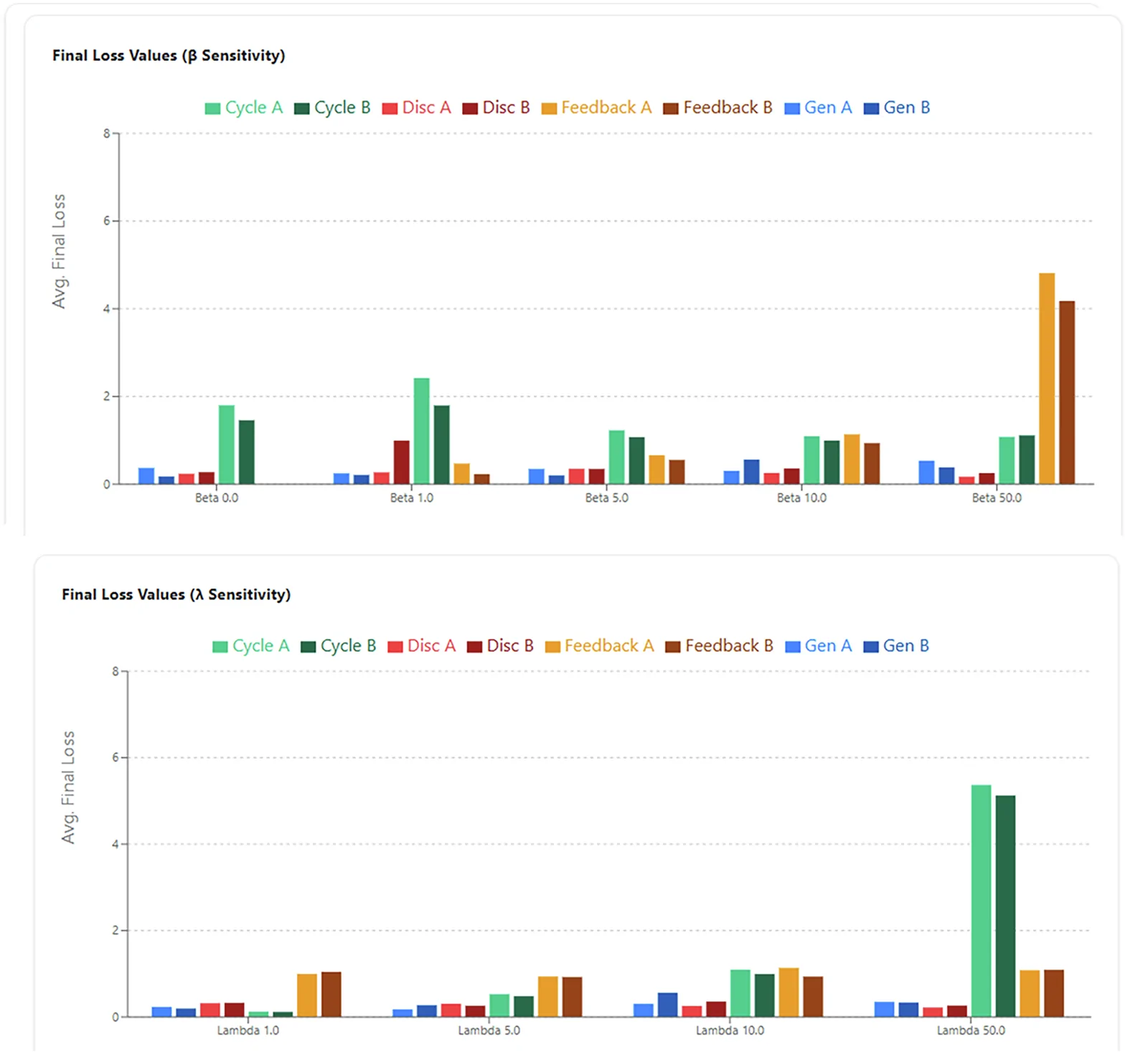
Sensitivity analysis of GANomics hyperparameters *β* and *λ*. Average validation losses at convergence for generator (Gen A/B), discriminator (Disc A/B), cycle-consistency (Cycle A/B), and feedback (Feedback A/B) components under varying **a** feedback loss weight *β* and **b** cycle-consistency weight *λ*, evaluated on the NB dataset. These results support the default choice of *β* = 10 and *λ* = 10 in GANomics.

To assess the robustness of GANomics to key hyperparameters, we conducted a sensitivity analysis of the feedback loss weight (*β*) and cycle-consistency weight (*λ*) using the NB dataset. We evaluated *β* ranged from [0, 1, 5, 10, 50] and *λ* ranged from [1, 5, 10, 50], where *β* = 0 corresponds to the standard CycleGAN without feedback loss. For each setting, we monitored validation losses of the generator, discriminator, cycle-consistency, and feedback components at convergence (Fig. 3).

As shown, moderate feedback loss weights (*β* ≈ 5–10) yielded the most balanced optimization across loss terms, whereas *β* = 0 lacked sample-level supervision, and large *β* (e.g., 50) led to instability due to over-dominance of the feedback objective. Similarly, *λ* values in the range 1–10 produced stable convergence, while *λ* = 50 overemphasized cycle consistency and disrupted the balance with adversarial and feedback losses. Based on these observations, we selected *β* = 10 and *λ* = 10 as default settings, providing a stable trade-off among competing objectives; the choice of *λ* is also consistent with the original CycleGAN formulation.

### Ablation analysis of feedback loss and cycle-consistency mechanisms

To disentangle the contribution of the feedback loss from the bidirectional cycle-consistency mechanism, we implemented two one-directional GAN baselines: MA → RS (G1) and RS → MA (G2), each trained using adversarial and feedback losses without cycle-consistency, and repeated 5 times for statistical analysis. Compared with the full GANomics model, these one-directional variants exhibited substantially higher validation losses and reduced stability. For example, the generator loss for MA → RS increased from 0.3898 (full model) to 0.7290 (G1), and for RS → MA from 0.5040 to 0.9817 (G2). Similarly, feedback alignment losses increased markedly (G1: 1.0778 vs. 7.8793; G2: 1.0144 vs. 5.9333). These results demonstrated that although feedback loss provides sample-level supervision, the bidirectional cycle-consistency mechanism plays a critical role in stabilizing training and improving translation fidelity. Together, these findings suggest that the performance gains of GANomics arise from the complementary effects of feedback supervision and cycle-consistent regularization.

### Sample size requirements for reliable microarray–RNA-seq translation with GANomics

To determine the number of paired samples required for reliable translation, we varied the training set size from 10 to 400 paired profiles, randomly selected from the full NB dataset (*n* = 498). The remaining samples were reserved for testing. To balance training efficiency and convergence, we used 50 epochs for larger training sets (100–400 pairs), 200 epochs for intermediate sizes (50–100 pairs), and 500 epochs for small training sets (10–50 pairs). Each train–test split was repeated five times with random sampling to ensure robustness. All other model hyperparameters were kept constant across experiments.

**Fig. 4:**
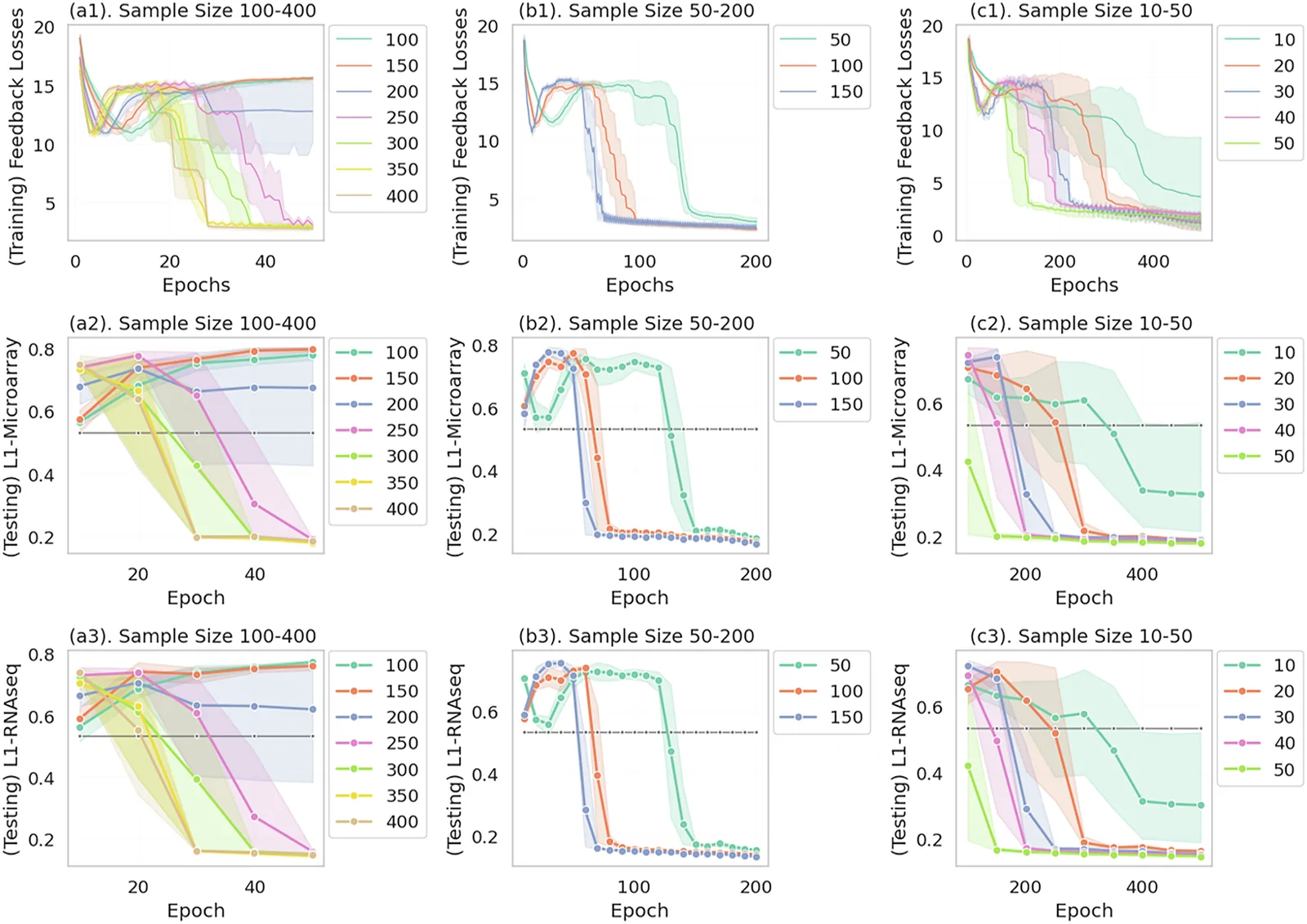
Influence of the training portion of the NB dataset on GANomics. **a** Sample size ranged from 100 to 400; **b** sample size ranged from 50 to 200; and **c** sample size ranged from 10 to 50. For each study, three results are presented as (1) feedback loss during the training process, (2) averaged L1-distance between real and synthetic Microarray profile, and (3) averaged L1-distance between real and synthetic RNA-seq profiles in the testing sets. The testing sets are the samples that have not been used in the GANomics training.

With 50-epoch, reliable convergence was observed only for ≥250 pairs, as indicated by stabilized losses and test-set L1 distances below the baseline (Fig. 4a). Increasing to 200 epochs reduced the threshold to ~50 pairs (Fig. 4b). Under an extended 500-epoch approach, a reliable model could be achieved with as few as 10 pairs, yielding test-set L1 distances well below the baseline (Fig. 4c). These results highlight a simple trade-off: fewer pairs require more training.

With 10 paired samples (named GANomics-10), we benchmarked against cross-platform or batch-effect methods (i.e., TDM, Quantile, ComBat, YuGene, and CuBlock) on the remaining 488 held-out NB samples, evaluating Pearson/Spearman correlations, L1/L2, MAE, and RMSE. GANomics-10 outperformed all others across metrics (p < 0.001, Table 1). CuBlock showed relatively high Spearman, consistent with its monotone and rank-preserving normalization, but lagged on error-based measures.

**Table 1:** GANomics compared to other translation algorithms, with (95% CI)

Algorithm	Pearson	Spearman	L1	L2	MAE	RMSE
GANomics (MA)	0.95 (0.94–0.95)	0.95 (0.95–0.95)	2436.91 (2407.76–2467.48)	32.44 (32.06–32.84)	0.24 (0.24–0.25)	0.32 (0.32–0.33)
TDM (MA)	0.72 (0.72–0.72)	0.73 (0.73–0.74)	5596.18 (5564.68–5626.57)	73.43 (73.07–73.78)	0.56 (0.55–0.56)	0.73 (0.73–0.74)
Quantile (MA)	0.72 (0.72–0.72)	0.73 (0.73–0.74)	5544.04 (5518.80–5568.81)	72.23 (71.91–72.54)	0.55 (0.55–0.55)	0.72 (0.72–0.72)
ComBat (MA)	0.65 (0.65–0.65)	0.65 (0.65–0.65)	6161.89 (6130.93–6191.81)	81.11 (80.73–81.47)	0.61 (0.61–0.62)	0.81 (0.81–0.81)
YuGene (MA)	0.73 (0.72–0.73)	0.73 (0.73–0.74)	7026.05 (7015.26–7036.91)	88.49 (88.39–88.58)	0.70 (0.70–0.70)	0.88 (0.88–0.88)
CuBlock (MA)	0.30 (0.28–0.31)	0.94 (0.94–0.95)	3841.77 (3705.17–3988.69)	711.51 (624.79–805.57)	0.38 (0.37–0.40)	7.10 (6.23–8.04)
GANomics (RS)	0.94 (0.94–0.94)	0.93(0.92–0.93)	2371.65 (2346.70–2397.61)	31.80 (31.45–32.14)	0.24 (0.23–0.24)	0.32 (0.31–0.32)
TDM (RS)	0.72 (0.72–0.72)	0.73 (0.73–0.74)	5153.56 (5126.11–5180.35)	67.70 (67.37–68.01)	0.51 (0.51–0.52)	0.68 (0.67–0.68)
Quantile (RS)	0.73 (0.73–0.73)	0.73 (0.73–0.74)	4751.34 (4721.92–4779.94)	66.70 (66.31–67.08)	0.47 (0.47–0.48)	0.67 (0.66–0.67)
ComBat (RS)	0.76 (0.76–0.76)	0.77 (0.77–0.77)	4799.27 (4775.56–4821.99)	63.49 (63.20–63.76)	0.48 (0.48–0.48)	0.63 (0.63–0.64)
YuGene (RS)	0.72 (0.72–0.72)	0.73 (0.73–0.74)	6021.85 (6010.44–6033.71)	84.69 (84.57–84.80)	0.60 (0.60–0.60)	0.85 (0.84–0.85)
CuBlock (RS)	0.32 (0.31–0.33)	0.95 (0.95–0.96)	2810.05 (2756.02–2867.06)	362.77 (340.37–387.98)	0.28 (0.27–0.29)	3.62 (3.40–3.87)

We next varied the number of paired samples (10–50) for GANomics. Across all sizes, performance exceeded the baseline defined by raw microarray-vs-RNA-seq pairs, with incremental gains as pairs increased—most notably lower L1/L2 (Table 2).

**Table 2:** Testing performance on GANomics models with different training size, with (95% CI)

Platform	Training size	Pearson	Spearman	L1	L2	MAE	RMSE
Baseline (MA vs RS)	-	0.72 (0.72–0.72)	0.73 (0.73–0.74)	5351.85 (5323.07–5379.72)	70.18 (69.84–70.52)	0.53 (0.53–0.54)	0.70 (0.70–0.70)
Synthetic MA	GANomics-10	0.95 (0.94–0.95)	0.95 (0.95–0.95)	2436.91 (2407.76–2467.48)	32.44 (32.06–32.84)	0.24 (0.24–0.25)	0.32 (0.32–0.33)
Synthetic MA	GANomics-20	0.94 (0.94–0.94)	0.93 (0.92–0.93)	2371.65 (2346.70–2397.61)	31.80 (31.45–32.14)	0.24 (0.23–0.24)	0.32 (0.31–0.32)
Synthetic MA	GANomics-30	0.96 (0.96–0.96)	0.96 (0.96–0.96)	2004.39 (1975.66–2033.66)	27.86 (27.47–28.26)	0.20 (0.20–0.20)	0.28 (0.27–0.28)
Synthetic MA	GANomics-40	0.96 (0.96–0.96)	0.96 (0.96–0.96)	1719.58 (1695.83–1744.50)	25.51 (25.15–25.88)	0.17 (0.17–0.17)	0.25 (0.25–0.26)
Synthetic MA	GANomics-50	0.96 (0.96–0.96)	0.96 (0.96–0.96)	1944.57 (1915.62–1974.89)	27.12 (26.76–27.49)	0.19 (0.19–0.20)	0.27(0.27–0.27)
Synthetic RS	GANomics-10	0.96 (0.96–0.96)	0.96 (0.96–0.96)	1647.27 (1627.33–1668.42)	24.55 (24.28–24.84)	0.16 (0.16–0.17)	0.25 (0.24–0.25)
Synthetic RS	GANomics-20	0.96 (0.96–0.96)	0.96 (0.96–0.97)	1941.88 (1910.49–1972.94)	27.43 (26.99–27.86)	0.19 (0.19–0.20)	0.27 (0.27–0.28)
Synthetic RS	GANomics-30	0.96 (0.96–0.97)	0.96 (0.96–0.97)	1591.95 (1565.18–1618.40)	24.03 (23.64–24.40)	0.16 (0.16–0.16)	0.24 (0.24–0.24)
Synthetic RS	GANomics-40	0.96 (0.96–0.96)	0.97 (0.97–0.97)	1803.05 (1771.75–1835.58)	25.72 (25.28–26.17)	0.18 (0.18–0.18)	0.26 (0.25–0.26)
Synthetic RS	GANomics-50	0.97 (0.97–0.97)	0.97 (0.97–0.97)	1514.89 (1491.77–1538.89)	23.19 (22.85–23.54)	0.15 (0.15–0.15)	0.23 (0.23–0.23)

**Fig. 5:**
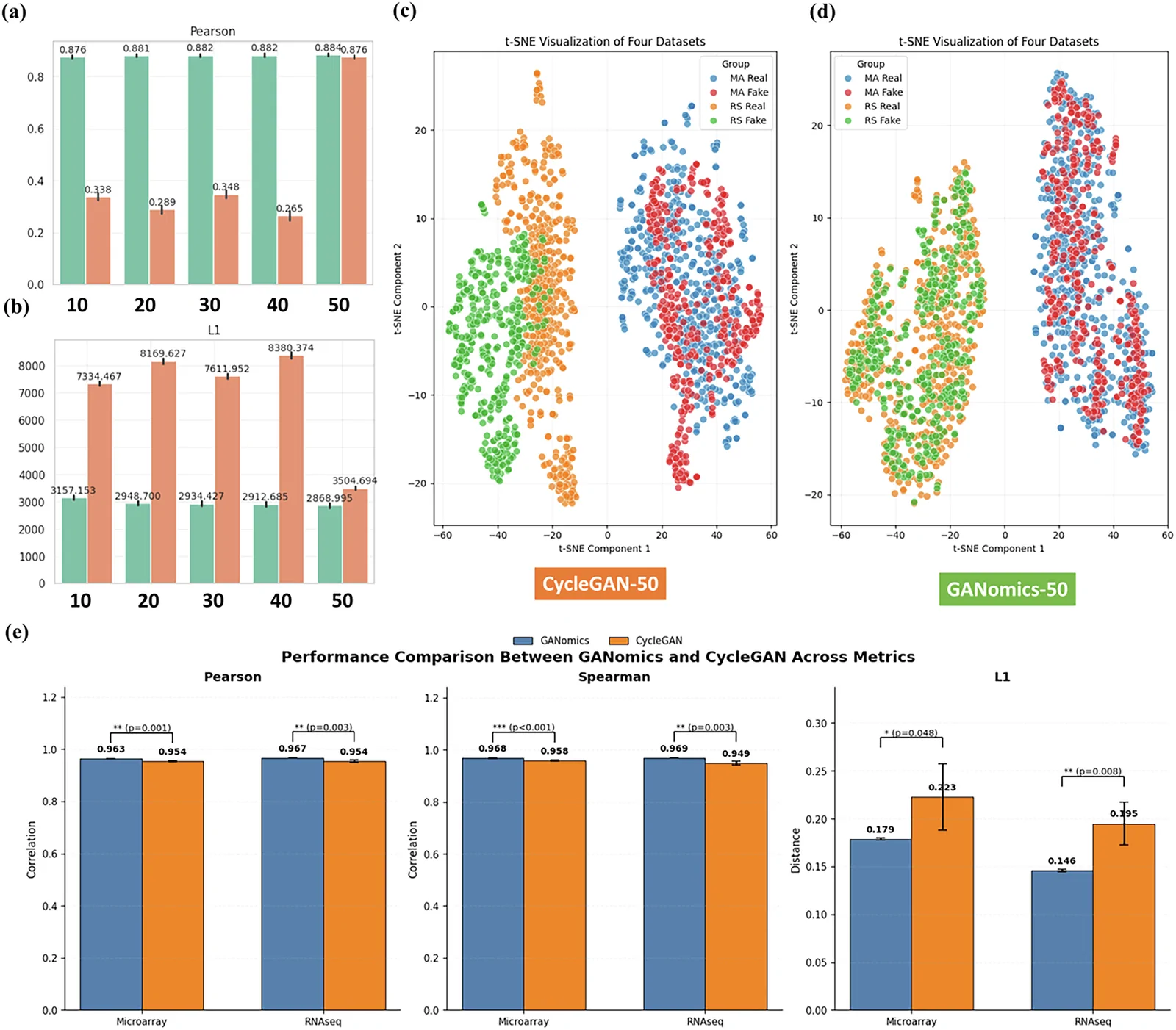
Quantitative and qualitative comparison between GANomics and CycleGAN across training sizes and evaluation metrics. **a** Pearson correlation and **b** L1 distance between real and synthetic profiles for GANomics and CycleGAN across training sizes (10–50 paired samples). GANomics consistently achieves higher correlation and lower reconstruction error across all sample sizes. **c**, **d** Representative t-SNE visualizations of real and synthetic testing samples using **c** CycleGAN-50 and **d** GANomics-50, showing improved alignment between real and synthetic distributions with GANomics. **e** Statistical comparison between GANomics-50 and CycleGAN-50 over five independent runs using identical train–test splits. GANomics achieves significantly higher Pearson and Spearman correlations and lower L1 distances in both translation directions (paired *t* test), confirming its consistent performance advantage over CycleGAN.

Since GANomics was built on CycleGAN, we also conducted an extensive comparison between GANomics and CycleGAN with identical training sizes (10–50). CycleGAN only converged at ≥50 pairs and still underperformed GANomics-50 on correlation and error metrics (Fig. 5a–c). t-SNE projections for one example of GANomics and CycleGAN with 30 and 50 training samples further showed real–synthetic co-localisation for GANomics but not CycleGAN (Fig. 5d–g). Based on this trade-off between data efficiency and accuracy, we used GANomics-50 for all subsequent NB analyses to assess biological preservation of synthetic data.

To further evaluate whether GANomics maintains an advantage when a moderate number of paired samples are available, we performed a statistical comparison between GANomics-50 and CycleGAN-50 across five independent random train–test splits, using identical splits for paired evaluation. For each run, we computed Pearson correlation, Spearman correlation, and L1 distance on the held-out test samples, and assessed significance using paired two-sided *t* tests (Fig. 5h). While the absolute differences between methods were smaller than in low-sample settings, GANomics consistently achieved higher correlations and lower L1 distances across both translation directions. For example, Pearson correlation improved from 0.954 to 0.963 (*p* = 0.001) for microarray translation and from 0.954 to 0.967 (*p* = 0.003) for RNA-seq translation, with similar improvements observed for Spearman correlation. L1 distances were also significantly reduced (*p* = 0.048 and *p* = 0.008 for the two directions, respectively). These results demonstrate that GANomics provides a consistent and statistically significant performance advantage over CycleGAN even when sufficient paired samples are available.

### GANomics generates expression profiles with high concordance

**Fig. 6:**
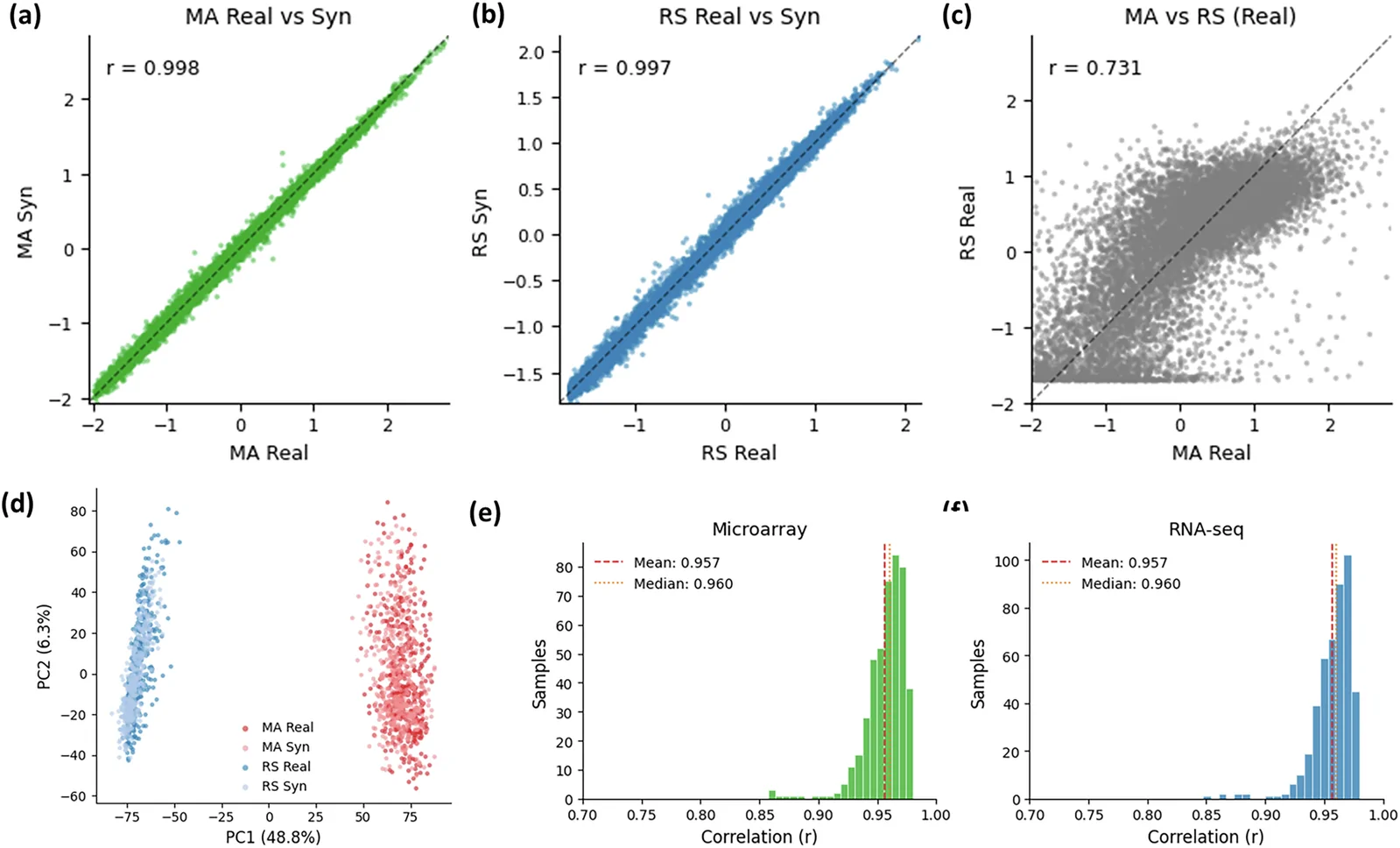
Real v.s Synthetic expression profiles generated by GANomics. **a**–**c** high concordance is observed between real and synthetic **a** microarray, and **b** RNA-seq expression profiles using GANomics translation, compared to the **c** baseline. **d** clustering analysis demonstrated that the synthetic expression profiles falls inside the feature space of real profiles in each platform. **e**, **f** Distribution of Pearson correlation among all testing samples in **e** Microarray and **f** RNA-seq platform, as per-sample correlations exceeded 0.96 in both platforms.

Using GANomics-50, we translated the remaining 448 NB samples (i.e., excluding the 50 used in training) in both directions and compared each synthetic profile to its real counterpart, yielding four datasets: microarray-real, microarray-synthetic, RNA-seq-real, and RNA-seq-synthetic. Mean expression across 10,042 genes showed tight agreement between real and synthetic on both platforms (Fig. 6a–c). A joint PCA of all 1792 profiles (448 × 4) placed synthetic samples co-localized with their real matches, indicating preservation of global structure (Fig. 6d). At the per-sample level, correlations exceeded 0.96 in both directions (Fig. 6e, f), demonstrating one-to-one translation across platforms.

### GANomics further generalizes across multiple datasets

We next assessed GANomics generalizability with five external datasets (i.e., TCGA-BRCA, TCGA-LUSC, TCGA-LAML, NCI-60, and METSIM). For each dataset, we developed a GANomics model with 50 paired samples (GANomics-50), and the remaining samples serve as a test set.

**Fig. 7:**
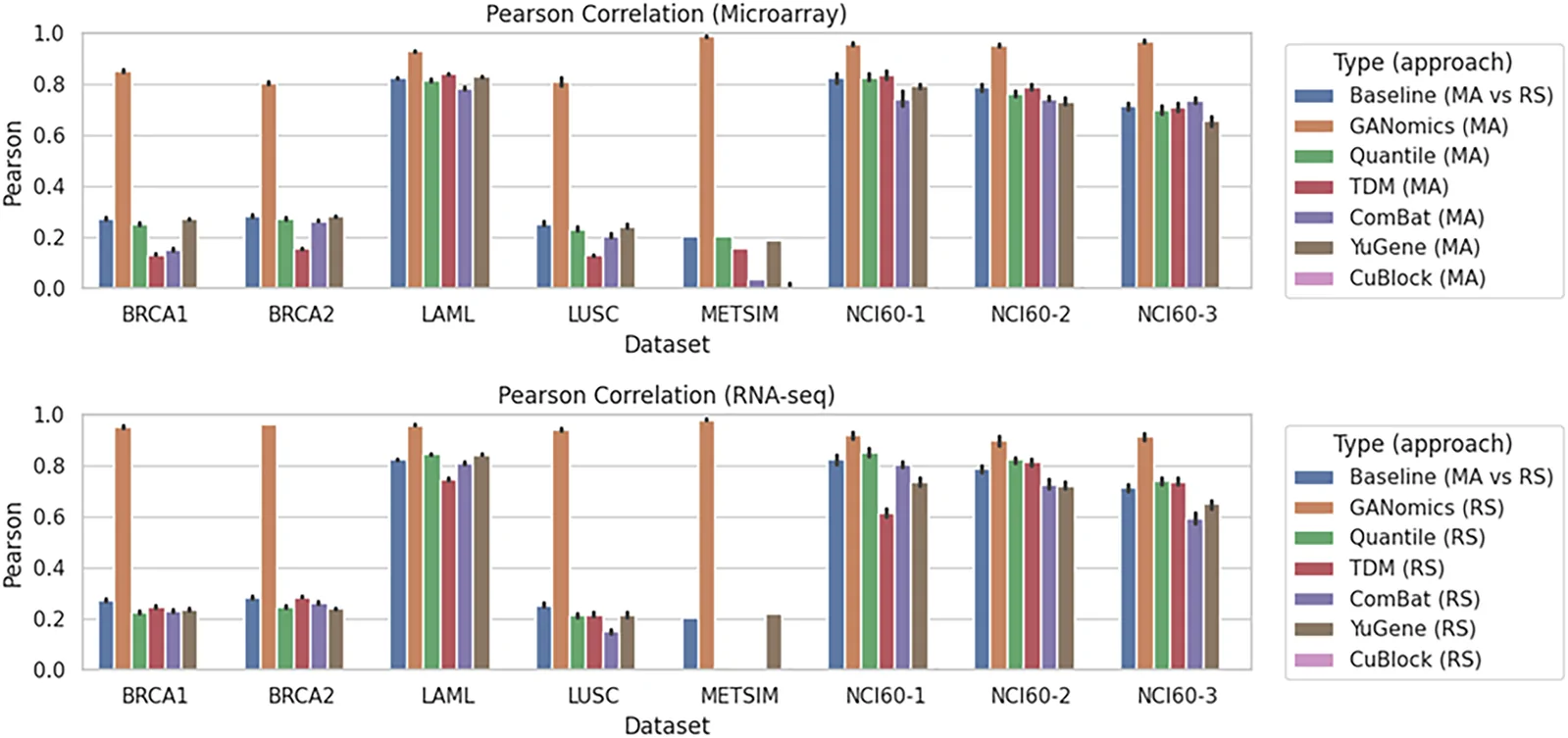
Cross-dataset generalization of GANomics on other benchmark datasets. The blue bar is the baseline, which reflects the correlation between the original Microarray and RNA-seq expression profiles from the paired samples. Orange bar represented GANomics performance; other approaches are represented as shown in the legend. Overall, GANomics outperformed other approaches across all cohorts and directions in both **a** microarray translation and **b** RNA-seq translation, by Pearson Correlation, consistent with the observations in NB experiments.

First, GANomics-50 models for all the datasets were benchmarked against TDM, Quantile, ComBat, YuGene, and CuBlock using per-sample comparisons alongside the baseline. Across datasets, GANomics achieved high Pearson/Spearman correlations between real and synthetic profiles and outperformed all comparators in both directions, including the baseline (Fig. 7). Some of these external datasets involved various platforms (e.g., three microarray platforms in NCI-60) and different quantification methods (e.g., two RNA-seq quantifications in BRCA), thus the consistency in performance indicates a potential diverse application of GANomics for various transcriptomic platforms and analysis methods. Full metric tables are provided in Table S2.

**Fig. 8:**
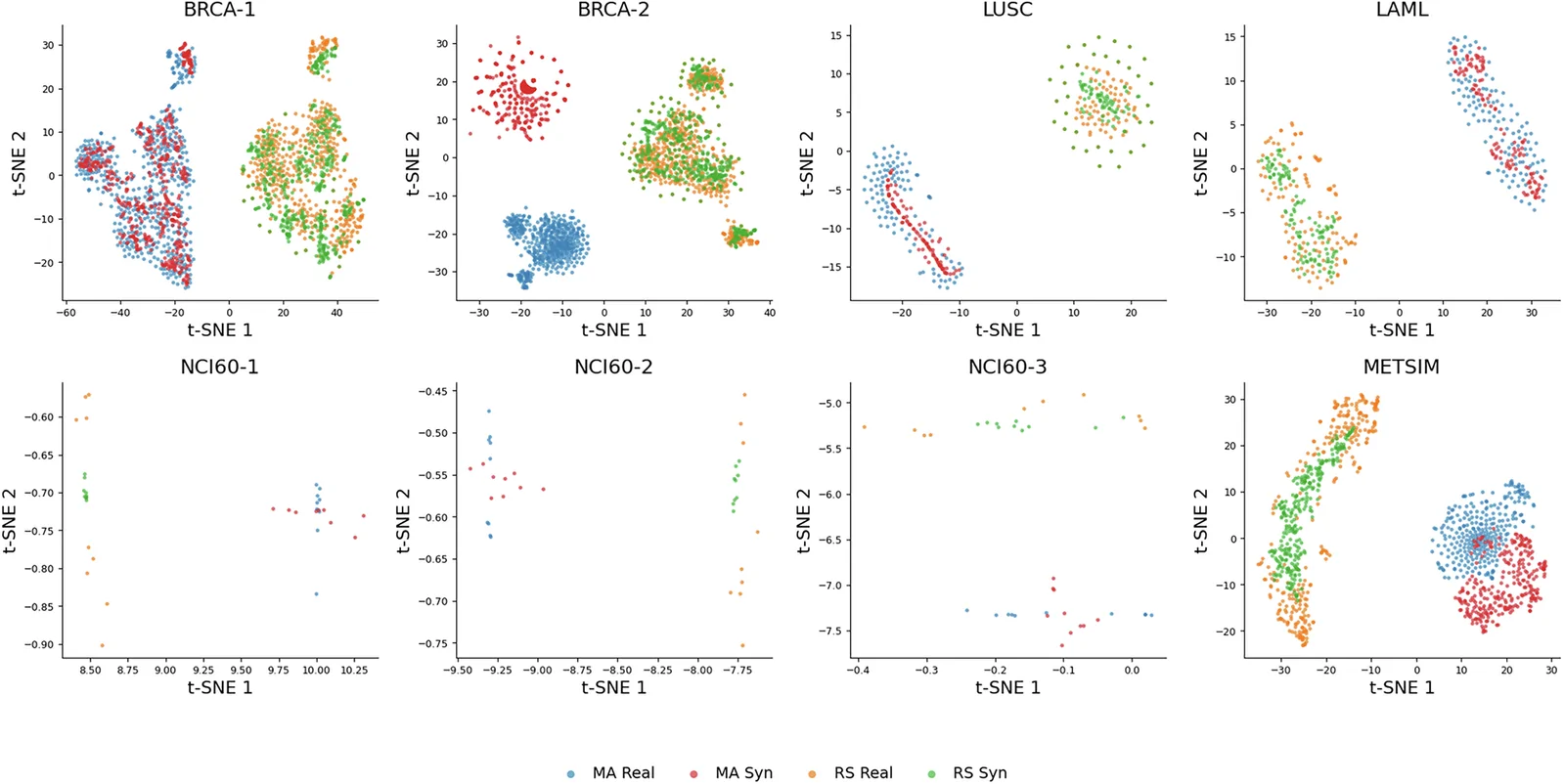
t-SNE analysis of real and synthetic expression profiles in testing samples for BRCA, NCI60 and METSIM datasets. Samples are visualized in different color by their category: blue: microarray real; red: Microarray synthetic; yellow: RNA-seq real; green: RNA-seq synthetic.

We further examined structure preservation via t-SNE over held-out testing samples (Fig. 8). For microarray to RNA-seq and RNA-seq to microarray, GANomics synthesized profiles co-localized with real profiles and separated cleanly from the source domain. Moreover, the translation between different Microarray platforms can be successfully translated to RNA-seq, as demonstrated in three NCI60 experiments. Similarly, from different RNA-seq platforms to Microarray, as demonstrated by two BRCA experiments. Besides applying GANomics-50, we also performed similar analyses on GANomics-10, and the t-SNE alignment of GANomics-10 resulted in a similar trend to transform data between two platforms, as provided in the supplementary file (Fig. S2).

**Fig. 9:**
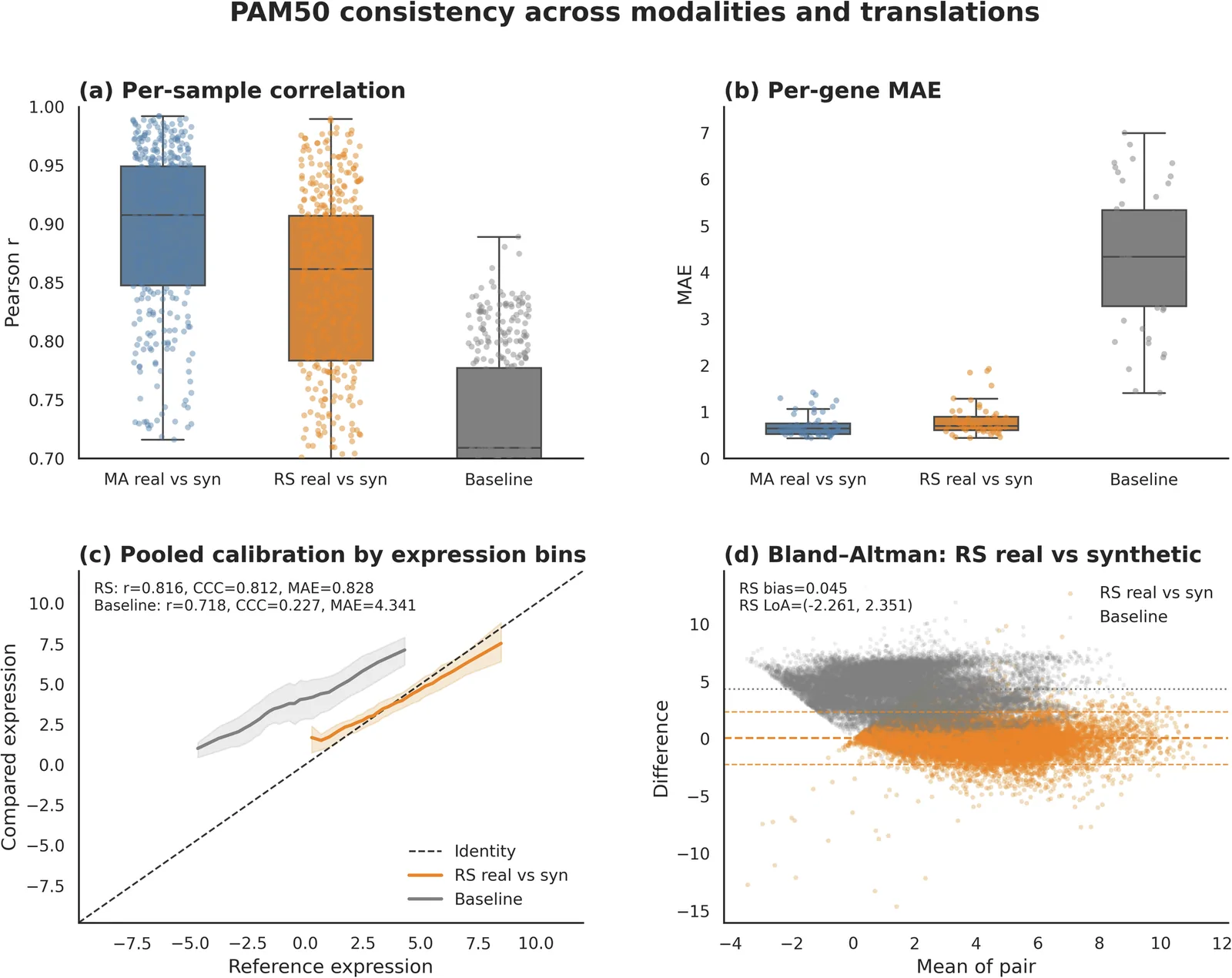
PAM50 gene expression consistency across modalities and translation strategies in the TCGA-BRCA (BRCA1) dataset. **a** Per-sample Pearson correlation across PAM50 genes comparing MA (real vs synthetic) and RS (real vs synthetic), and the baseline (microarray vs RNA-seq). **b** Per-gene mean absolute error (MAE) across samples for the same comparisons. **c** Binned calibration curves showing the relationship between real and compared expression values across expression ranges. Shaded regions represent interquartile ranges within bins. **d** Bland–Altman analysis for RNA-seq real vs synthetic profiles, with the baseline overlaid for comparison.

To demonstrate the clinical relevance of GANomics, we evaluated the preservation of the PAM50 gene signature for breast cancer classification in the TCGA-BRCA dataset (Fig. 9). Compared with the cross-platform baseline (microarray vs RNA-seq), GANomics translations showed substantially higher per-sample correlations and lower per-gene MAE, indicating improved agreement at both the sample and gene levels (Fig. 9a, b). At the expression level, binned calibration analysis revealed that GANomics-translated profiles closely followed the identity relationship across the dynamic range, whereas the baseline exhibited systematic deviations, particularly at higher expression values (Fig. 9c). Consistently, Bland–Altman analysis demonstrated reduced bias and narrower limits of agreement for GANomics compared with the baseline, indicating improved sample-level consistency (Fig. 9d). These results confirm that GANomics not only improves statistical alignment between platforms but also preserves clinically relevant gene expression patterns, supporting its utility for maintaining established molecular signatures across transcriptomic technologies.

### GANomics preserves DEGs and pathways

To assess whether synthetic profiles preserve biologically meaningful signal, we performed differential expression analysis on the NB hold-out set translated with GANomics-50. Among the 448 held-out samples, 242 had clinical outcomes (Favorable: event-free ≥1000 days without chemotherapy; Unfavorable); DEGs were computed between these two groups for each platform/dataset: Microarray-real, Microarray-synthetic, RNA-seq-real, and RNA-seq-synthetic.

**Fig. 10:**
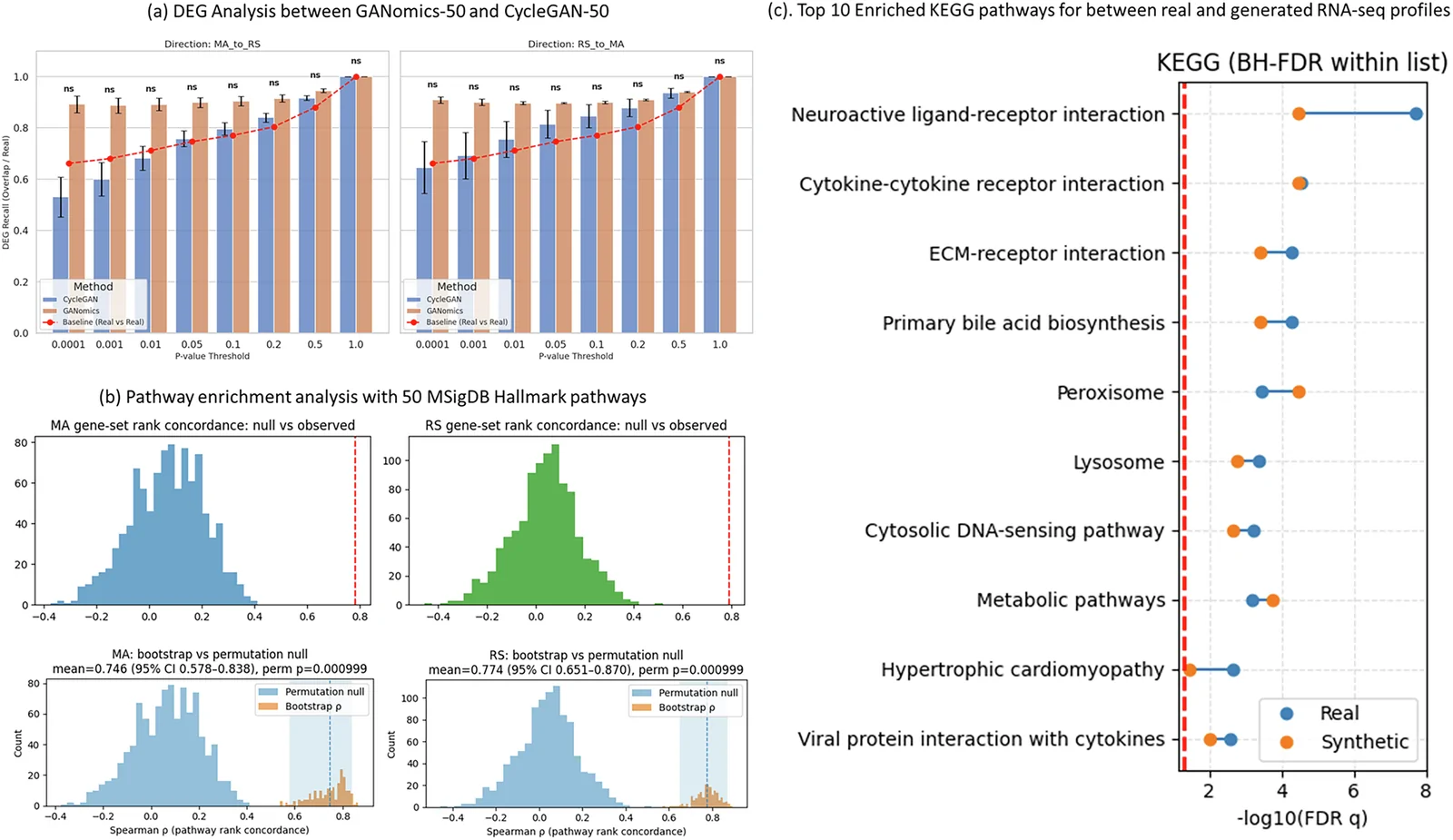
Preservation of biological signals in GANomics-translated profiles compared with CycleGAN. **a** Differentially expressed gene (DEG) overlap between real and synthetic profiles across a range of *p*-value thresholds for microarray→RNA-seq (left) and RNA-seq→microarray (right) translations. The red dashed line indicates the baseline overlap between real microarray and RNA-seq profiles. Statistical significance between methods is indicated (ns: not significant). **b** Pathway-level concordance using MSigDB Hallmark gene sets. The top panels show permutation null distributions of Spearman rank correlation (*ρ*) between real and synthetic pathway significance, with observed *ρ* (red dashed line) lying far from the null. Bottom panels show bootstrap distributions (orange) overlaid with permutation null (blue), demonstrating robust and significant pathway concordance for both microarray and RNA-seq (*P* < 0.001). **c** KEGG pathway enrichment comparison between real and GANomics-generated RNA-seq profiles. Top enriched pathways identified in real data are consistently recovered in synthetic data, with similar significance levels (−log10 FDR *q*). The red dashed line indicates the significance threshold (FDR *q* = 0.05).

Across platforms, the top-ranked DEGs showed substantial concordance between real and synthetic data, with ~60% overlap in both microarray and RNA-seq analyses—well above random expectation (Fig. 10a, red dotted line). To contextualize this biological fidelity, we compared GANomics with a representative baseline, CycleGAN, using the same training configuration (50 paired samples). GANomics consistently achieved higher DEG overlap with real data across a range of significance thresholds, particularly at more stringent levels (e.g., p < 0.05), whereas CycleGAN showed reduced concordance, indicating weaker preservation of phenotype-associated, sample-level signals (Fig. 10a). We focused on CycleGAN as a representative generative baseline for DEG comparison, as normalization-based methods (e.g., ComBat, QN) largely preserve gene rankings and therefore do not meaningfully alter DEG sets, making such comparisons uninformative.

We next evaluated pathway-level conservation using MSigDB Hallmark enrichment on effect-size–ranked genes. To establish a null, we permuted Hallmark gene membership on the synthetic side (size-matched) and recomputed enrichment. Observed Spearman rank concordance (*ρ*) between real and synthetic pathway significance lay far to the right of the permutation null (Fig. 10b). Bootstrap resampling (80% of samples, *B* = 200) yielded *ρ* = 0.75 (95% CI: 0.58–0.84) for microarray and *ρ* = 0.77 (95% CI: 0.65–0.87) for RNA-seq, each *P* < 0.001 versus the null. Top-K overlaps were likewise high (mean Jaccard ≈0.68 for Top-10 and ≈0.64 for Top-20), exceeding chance expectation.

For biological context, the top KEGG pathways identified from real RNA-seq remained significant in the synthetic RNA-seq (Fig. 10c; dashed line, *P* = 0.05), with similar trends observed for microarray. Notably, pathways such as neuroactive ligand–receptor interaction, cytokine–cytokine receptor interaction, ECM–receptor interaction, and Peroxisome were consistently recovered, aligning with known NB biology.

These results demonstrate that GANomics preserves gene-level and pathway-level biological signals, not merely improving statistical alignment metrics but maintaining biologically interpretable structure in translated profiles, outperforming both generative and normalization-based baselines.

### GANomics enables cross-platform predictive classifiers

We evaluated the utility of GANomics-translated profiles for downstream predictive modeling under two practical scenarios: (Q1) whether a classifier trained on real data can accurately predict labels using synthetic profiles, and (Q2) whether a classifier trained on synthetic data can generalize to real samples. Scenario 1 reflects the ability to extend established assays across platforms (e.g., applying microarray-based models to RNA-seq data), whereas Scenario 2 assesses whether models developed from legacy data can be transferred to newer technologies.

To systematically assess these scenarios, we split the 498 NB samples into two disjoint sets—one for training GANomics and the other for classifier development and evaluation—and repeated this procedure for models trained with 10, 20, 50, or 100 paired samples. For example, in GANomics-50, the remaining 448 samples were used for classification, of which 242 had clinical outcome labels (favorable/unfavorable). For each platform, we constructed four datasets: Train-real, Test-real, and their translated counterparts train-synthetic and test-synthetic.

**Fig. 11:**
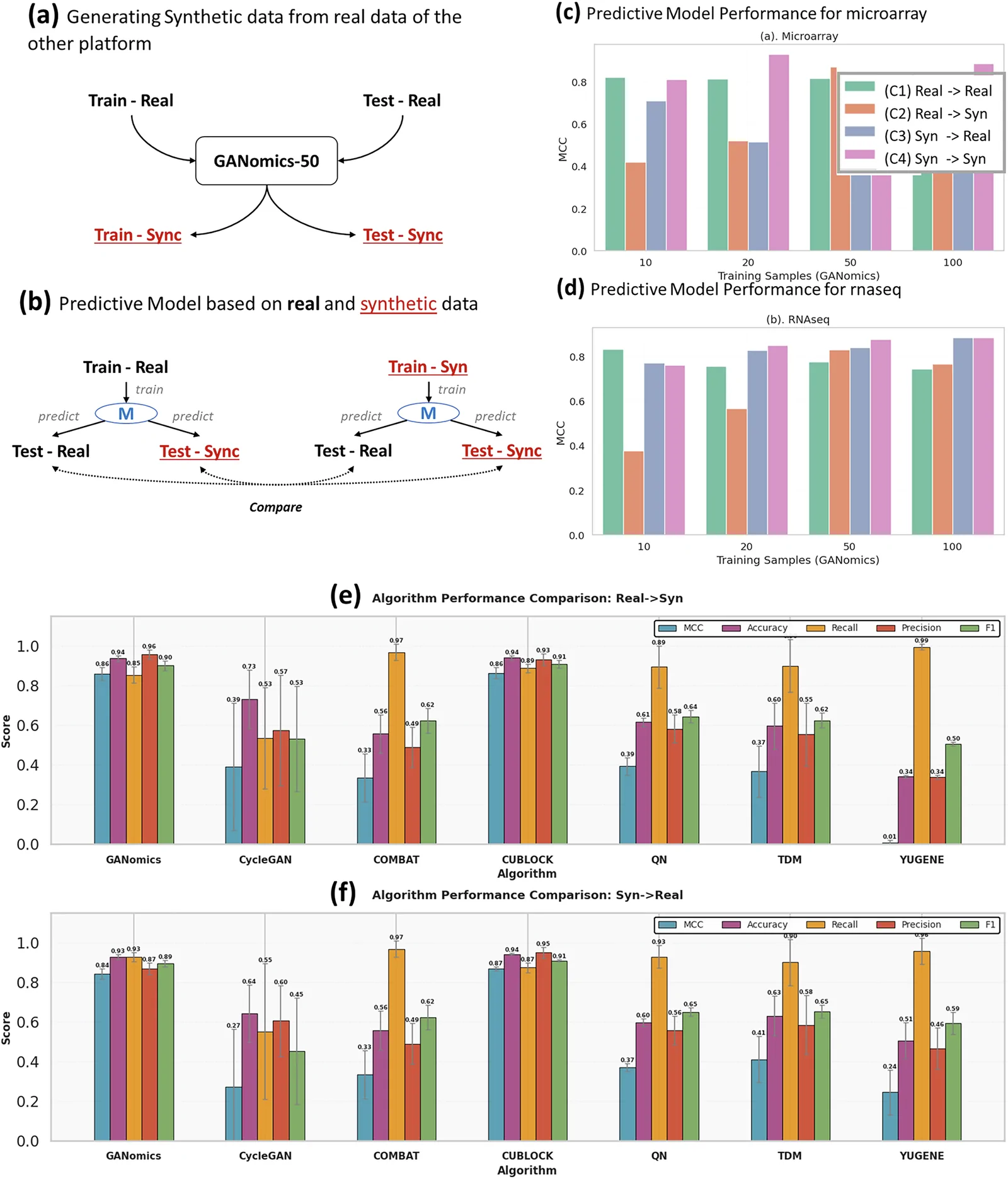
Evaluation of cross-platform predictive modeling using GANomics-translated profiles and comparison with baseline methods. **a** Schematic of data synthesis using GANomics, where real training and testing samples are translated to generate corresponding synthetic datasets. **b** Classification framework illustrating four evaluation configurations: C1 Real→Real (baseline), C2 Real→Synthetic, C3 Synthetic→Real, and C4 Synthetic→Synthetic. **c**, **d** Predictive performance (MCC) of Random Forest classifiers across different GANomics training sizes (10, 20, 50, and 100 paired samples) for **c** microarray and **d** RNA-seq domains. **e**–**f** Comparison of predictive performance across translation and normalization methods, including GANomics, CycleGAN, ComBat, CuBlock, QN, TDM, and YuGene, evaluated under **e** Real→Synthetic and **f** Synthetic→Real scenarios.

We evaluated four configurations (Fig. 11a, b): C1 Real→Real (baseline, denotes training and testing within the same platform), C2 Real→Synthetic (tests Q1), C3 Synthetic→Real (tests Q2), and C4 Synthetic→Synthetic. Random Forest (RF) classifiers were trained and evaluated separately in microarray and RNA-seq spaces, with Matthews correlation coefficient (MCC) as the primary metric (Fig. 11c, d) and additional metrics reported in Table S1.

To further assess practical utility, we compared predictive performance across multiple translation methods, including GANomics, CycleGAN, CuBlock, TDM, YuGene, QN, and ComBat (Fig. 11e, f). GANomics-50 consistently achieved strong and stable performance across all configurations, whereas CycleGAN and normalization-based approaches showed reduced predictive accuracy. Although CuBlock demonstrated competitive performance in some scenarios, it exhibited substantially poorer alignment between real and translated profiles, consistent with its higher reconstruction error and weaker correlation metrics. These results indicate that GANomics preserves not only global expression distributions but also biologically meaningful, sample-level information critical for prediction.

Performance trends were strongly dependent on the number of paired samples used to train GANomics. With 10–20 pairs, performance declined from C1 to C2/C3, reflecting limited translation accuracy in low-data settings. As the training size increased to ≥50 pairs, this gap narrowed substantially: C2 and C3 approached C1, indicating reliable cross-platform transfer in both directions. Notably, C4 (Synthetic→Synthetic) was comparable to, and occasionally exceeded, C1, suggesting that translation may reduce nuisance variation and yield cleaner decision boundaries. Overall, GANomics-50 provided the best balance between data efficiency and predictive accuracy and was therefore used for downstream analyses.

To further examine error structure, we analyzed misclassifications under GANomics-50 (Table 3). Most false positives and false negatives were shared across C1–C4, indicating that the same inherently ambiguous samples remain difficult to classify regardless of whether real or synthetic data are used. Concordance was highest between C1 and C4, supporting the feasibility of training and deploying classifiers entirely in the translated space. As expected, C3 (Synthetic→Real) introduced some additional false positives, consistent with a mild domain shift when applying models trained on synthetic data to real profiles.

**Table 3:** Wrongly predicted samples analysis in GANomics-50

Type	Classifier configuration	Recall	Precision	MCC	False positives*	False negatives*
Microarray	**C1 Real-Real (baseline)**	0.905	0.864	0.816	1, 72, 75, 78, 80, 112	37, 52, 63, 100
	**C2 Real-Syn**	0.857	0.973	0.870	1	24, 25, 37, 52, 63, 100
	**C3 Syn-Real**	0.952	0.755	0.749	1, 44, 47, 48, 56, 57, 58, 75, 78, 80, 97, 108, 112	24, 63
	**C4 Syn-Syn**	0.905	0.905	0.851	1, 41, 78, 112	25, 37, 52, 63
RNA-seq	**C1 Real-Real (baseline)**	0.857	0.857	0.776	1, 72, 75, 78, 80, 112	24, 25, 37, 63, 82, 100
	**C2 Real-Syn**	0.857	0.923	0.831	1, 72, 75	24, 25, 37, 52, 63, 100
	**C3 Syn-Real**	0.952	0.851	0.840	57, 72, 75, 79, 80, 93, 108	24, 63
	**C4 Syn-Syn**	0.976	0.872	0.876	1, 72, 75, 78, 79, 80	63

^*^The numbers represent sample IDs which were auto-generated during the analysis, consistent across classifier configurations, but do not necessarily match the original sample ID.

### GANomics improves predictive modeling through data augmentation

To directly assess whether integrating data across platforms improves predictive performance beyond using a single modality alone, we evaluated the extent to which GANomics-generated synthetic profiles can augment real training data. In addition to real-only training (Real→Real), we considered two combined training strategies: (1) averaged integration, in which real and synthetic profiles from the same sample were combined by element-wise averaging, and (2) stacked augmentation, in which real and synthetic samples were jointly included to expand the training set. In both cases, synthetic profiles were generated by translating training samples from the alternate platform (i.e., microarray→RNA-seq or RNA-seq→microarray), thereby enabling explicit cross-platform data integration within a unified feature space.

**Fig. 12:**
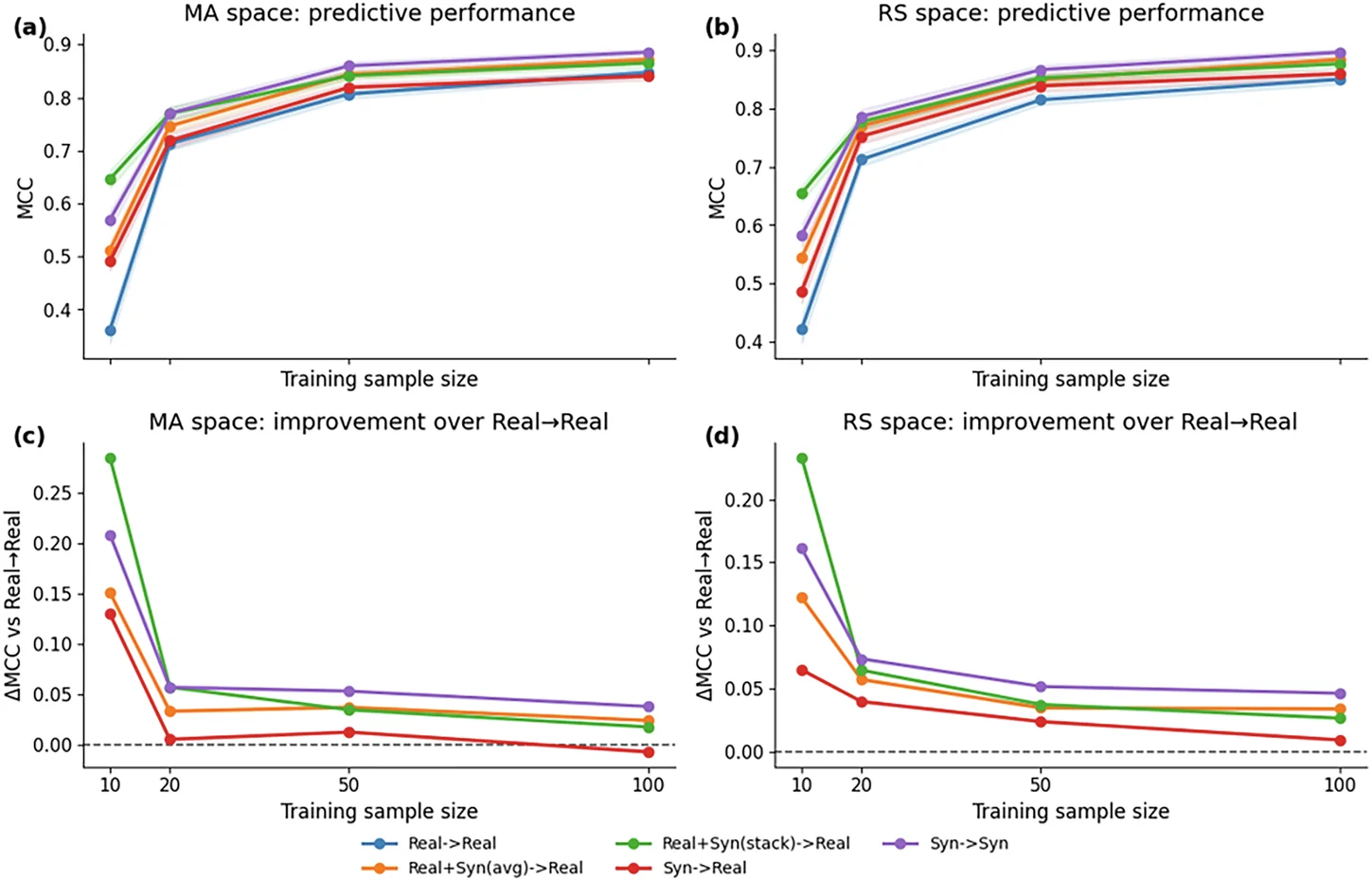
Impact of incorporating GANomics-generated synthetic data on predictive modeling across training sample sizes. **a**, **b** Predictive performance (MCC) of Random Forest classifiers in **a** microarray (MA) and **b** RNA-seq (RS) spaces under different training strategies, including real-only (Real→Real), synthetic-only (Syn→Real, Syn→Syn), and combined training using averaged (Real+Syn(avg)→Real) or stacked augmentation (Real+Syn(stack)→Real). **c**, **d** Improvement in MCC relative to the Real→Real baseline (ΔMCC) for **c** MA and **d** RS spaces. Positive values indicate performance gains from incorporating synthetic data. These results indicate that GANomics-generated synthetic data are most beneficial in low-data regimes, where they effectively increase training sample size and improve model generalization.

As shown in Fig. 12, incorporating synthetic data led to consistent improvements over real-only training, particularly in low-sample regimes (e.g., *N* = 10–20). Notably, stacked augmentation (Real+Syn(stack)→Real) achieved the best performance across both microarray and RNA-seq spaces, indicating that translated profiles provide complementary information beyond simple denoising or averaging effects. Averaged integration also improved performance in some settings, although its impact was generally smaller than that of stacking. Improvements from combined training—especially for stacked augmentation—were statistically significant in low-sample settings (*p* < 0.05; Supplementary Table S3). These results demonstrate that legacy data, when translated into the target platform using GANomics, can meaningfully enhance predictive modeling performance.

Importantly, the benefit of incorporating synthetic data diminished as training sample size increased, with all methods converging at larger sample sizes (*N* ≥ 100). This pattern suggests that GANomics-generated profiles are most valuable in limited-data scenarios, where they effectively increase the usable sample size and improve model robustness. Moreover, because this augmentation framework can be applied in both directions (microarray→RNA-seq and RNA-seq→microarray), it supports flexible reuse of legacy datasets as well as compatibility with existing platform-specific models.

## Discussion

The evolution of transcriptomic approaches is inevitable due to the advancement in technologies. Come with it is a challenge in integrating legacy data with data from newer technologies. Microarrays, which dominated the field for over two decades, generated vast repositories of clinically and biologically valuable data. However, the shift to RNAseq has rendered much of this legacy data under-utilized due to platform incompatibility.

To address these challenges, we developed GANomics, a generative framework for translating transcriptomic profiles across platforms. GANomics introduced a paired-aware generative translator that was successfully trained on as few as 10–50 pairs of profiles that can bridge legacy microarrays with RNA-seq. Unlike dataset-level harmonizers (ComBat, QN) or unpaired CycleGAN variants, GANomics jointly optimized per-sample consistency and platform-wide alignment, enabling bidirectional translation that preserves genes, pathways, and downstream predictive utility. Across NB, NCI-60, TCGA, and METSIM, GANomics converts historical data into RNA-seq-like profiles (and vice versa) without compromising biological signal and allowing classifier development and deployment directly on translated profiles.

Notably, predictive classifiers trained on GANomics-generated (i.e., synthetic) data performed comparably to those trained on real data, especially when 50 paired samples were used to train GANomics. This indicates that GANomics has the potential to reduce extensive real-world profiling, offering a cost-effective alternative for training machine learning models. Moreover, the use of synthetic data reduced noise and maintained misclassification patterns consistent with real data, suggesting robustness and generalizability.

Although GANomics is designed as a bidirectional framework, its practical value lies in enabling interoperability between transcriptomic platforms. In one direction (microarray → RNA-seq), GANomics allows large legacy microarray datasets with rich clinical annotations to be translated into RNA-seq–like profiles, enabling their integration with modern cohorts and effectively increasing usable sample size. In the reverse direction (RNA-seq → microarray), GANomics enables continued use of clinically validated microarray-based signatures (e.g., MammaPrint) after a transition to RNA-seq technologies. In such cases, RNA-seq profiles can be translated into microarray-like representations, allowing direct application of established diagnostic models without the need for costly revalidation. These use cases highlight that bidirectional translation is not only a technical feature but also a practical mechanism for preserving and extending the value of existing biomedical resources.

Although CycleGAN achieves comparable performance to GANomics in terms of global correlation metrics (i.e., expression value alignments) when ≥50 paired samples are available, this similarity primarily reflects alignment at the distribution level rather than preservation of sample-specific biological signals. GANomics was explicitly designed to address this limitation through the pair-aware feedback loss, which constrains each generated profile to match its paired counterpart. As a result, GANomics maintains biologically meaningful variation at the individual sample level, which is critical for downstream applications. This distinction is evident in predictive modeling experiments, where classifiers trained on CycleGAN-translated data (*β* = 0) exhibit degraded performance, particularly in cross-domain settings (e.g., Real → Synthetic and Synthetic → Real), whereas GANomics preserves predictive accuracy across configurations (Supplementary Fig. S3). These findings suggest that while CycleGAN may be sufficient for coarse distribution alignment when large, paired datasets are available, GANomics provides a practical advantage in applications that require faithful preservation of biological signals, such as biomarker transfer, clinical prediction, and translational modeling. Therefore, GANomics is particularly preferable when sample-level fidelity and downstream utility are critical, even in settings with moderate paired sample sizes.

While our proof-of-concept focused on paired microarray and RNA-seq data, the framework is broadly applicable. One of the potential applications is in systems biology by providing a computational approach for translating transcriptomic profiles between cell types or experimental conditions, thus maximizing the utility of historical or incomplete datasets. For example, in large-scale pharmacogenomics initiatives such as the LINCS program, re-profiling thousands of compounds across evolving technologies or diverse biological systems is impractical. GANomics supports bidirectional translation across platforms, helping extend the value of existing datasets and reducing the need for repeated experiments. Future directions include expanding GANomics to integrate additional omics layers (e.g., proteomics, metabolomics) and validating performance in other disease areas. These efforts will enhance multi-modal data integration, enabling more comprehensive modeling of complex biological processes.

While GANomics demonstrates strong performance in translating between microarray and RNA-seq platforms within a given cohort, the current study focuses on isolating platform-specific transformations rather than inter-cohort variability. Generalization to independent cohorts introduces additional challenges, including batch effects and biological heterogeneity. Although our evaluation across multiple datasets suggests robustness of the framework, future work is needed to systematically assess cross-cohort generalization, potentially by integrating batch correction with platform translation. This represents an important direction for extending GANomics to broader real-world applications.

In summary, GANomics offers a robust generative AI framework for transcriptomic profile translation, effectively bridging the technological gap between legacy and modern platforms. Our findings demonstrate that as few as 10 paired samples are effective to develop the GANomics to transform gene expression profiles between microarrays and RNA-seq, where using 50 paired samples is sufficient to train a reliable model that supports both developing a reliable predictive classifier and preserving biological signal. By enabling cross-platform translation and maintaining biological fidelity, GANomics facilitates the reuse of historical data, reduces the need for redundant experimentation, and supports transcriptomic innovation. As AI continues to reshape biomedical research, GANomics represents a practical and data-efficient approach for integrative analysis and translational genomics.

## Methods

### Implementation of GANomics

The most innovative part of GANomics is to extend CycleGAN^30^ by adding a new, paired-aware loss term (i.e., feedback loss) that regresses generated outputs to their known paired counterparts. In the meantime, CycleGAN’s two existing losses for bidirectional adversarial and cycle-consistency objectives are preserved.

In detail, G_MA→SEQ_ (G1) predicts RNA-seq based on Microarray expression profiles, while G_SEQ→MA_ (G2) predicts microarrays based on RNA-seq gene expression profiles. Both generators were autoencoders with dense layers suitable to handle transcriptomics data^25,27^. In practice, we use 1 × 1 convolutions to emulate fully connected layers over gene features; this behaves as a dense mapping over the feature axis. With that setting, GANomics was built on CycleGAN with three different types of loss functions. These are adversarial losses, cycle consistency losses, and feedback losses.

Adversarial loss (L_GAN_) in GANomics follows the CycleGAN framework and is designed to align the distribution of regenerated profiles with that of the target domain. The goal of the generator **G1** is to produce synthetic profiles that resemble real samples from the target domain, while the discriminator **D**_**y**_ learns to distinguish between true target sample y and generated sample **G1**(x)^31^. Similarly, the generator **G2** and discriminator **D**_**x**_ operate in the opposite direction. The adversarial training objective is therefore to let the generators minimize the loss to fool the discriminators, while the discriminators maximize the loss to correctly classify real versus generated samples. As expressed as (Eq. 1).

$${{\mathscr{L}}}_{{\rm{GAN}}}\left({\rm{G}}1,{{\rm{D}}}_{{\rm{Y}}},{\rm{X}},{\rm{Y}}\right)={{\mathbb{E}}}_{{\rm{y}}\sim {{\rm{p}}}_{{\rm{data}}}\left({\rm{y}}\right)}\left[\log {{\rm{D}}}_{{\rm{Y}}}\left({\rm{y}}\right)\right]+{{\mathbb{E}}}_{{\rm{x}}\sim {{\rm{p}}}_{{\rm{data}}}\left({\rm{x}}\right)}\left[\log \left({1-{\rm{D}}}_{{\rm{Y}}}\left({\rm{G}}1\left({\rm{x}}\right)\right)\right)\right]$$

1 $${{\mathscr{L}}}_{{\rm{GAN}}}\left({\rm{G}}2,{{\rm{D}}}_{{\rm{x}}},{\rm{X}},{\rm{Y}}\right)={{\mathbb{E}}}_{{\rm{x}}\sim {{\rm{p}}}_{{\rm{data}}}\left({\rm{x}}\right)}\left[\log {{\rm{D}}}_{{\rm{x}}}\left({\rm{x}}\right)\right]+{{\mathbb{E}}}_{{\rm{y}}\sim {{\rm{p}}}_{{\rm{data}}}\left({\rm{y}}\right)}\left[\log \left({1-{\rm{D}}}_{{\rm{Y}}}\left({\rm{G}}2\left({\rm{y}}\right)\right)\right)\right]$$

where **G1: X** → **Y** translates microarray profiles into RNA-seq space, and **G2: Y** → **X** translates RNA-seq into microarray space. Together, these objectives enforce that generated profiles are indistinguishable from real data in each domain.

Cycle consistency losses (L_CYC_)^30^ from CycleGAN ensures that for each *x* from domain *X*, the translation cycle should be able to bring *x* back to the original domain, i.e., *x* → *G1*(*x*) → *G2*(*G1*(*x*)) ≈ *x*. Similarly, for each image *y* from domain *Y*, *G1* and *G2* should also satisfy backward cycle consistency: *y* → *G2*(*y*) → *G1*(*G2*(*y*)) ≈ *y*. The cycle consistency loss used in this study was described in (Eq. 2), where x and y are the real X and Y, and G2(G1(x)) and G1(G2(y)) are the regenerated X and Y, respectively.

2 $${{\mathscr{L}}}_{{CYC}}\left(G1,G2\right){\mathbb{=}}{\mathbb{E}}\left[{||G}2\left(G1\left(x\right)\right)-{x||}\right]{\mathbb{+}}{\mathbb{E}}\left[{||G}1\left(G2\left(y\right)\right)-{y||}\right]$$

Feedback loss is to steer the network in the direction of the correct generation of profiles. The feedback loss measures the difference between the G1(x) (i.e., Gen. Y) and the y paired to real x (i.e., Paired Y), as well as the G2(y) (i.e., Gen. X) and the x paired to real y (i.e., Paired X), as illustrated in (Eq. 3).

3 $${{\mathscr{L}}}_{{FB}}\left(G1,G2,P\right)={{\mathbb{E}}}_{(x,y)\in p}\left[{||}G1\left(x\right)-{y}_{{paired}}{||}+{||}G2\left(y\right)-{x}_{{paired}}{||}\right],$$

By combining three loss functions, the full objective of GANomics is illustrated in (Eq. 4), Where *λ* and *β* control the two goals’ relative priority.

4 $${\mathscr{L}}\left(G2,{G1,D}_{X},{D}_{Y}\right)={{\mathscr{L}}}_{{GAN}}\left(G1,{D}_{Y},X,Y\right)+{{\mathscr{L}}}_{{GAN}}\left(G2,{D}_{X},Y,X\right)+\lambda {{\mathscr{L}}}_{{cyc}}\left(G1,G2\right)+\beta {{\mathscr{L}}}_{{fb}}\left(G1,G2,P\right)$$

After all, we aim to solve (Eq. 5).

5 $${{\rm{G}}}^{* },{{\rm{D}}}^{* }=\mathop{{\rm{\arg }}\min }\limits_{G1,G2}\mathop{\max }\limits_{{D}_{X},{D}_{Y}}{\mathscr{L}}\left(G1,G2,{D}_{X},{D}_{Y}\right)$$

In practice, each iteration draws one paired MA–RS mini-batch for the feedback term and one unpaired mini-batch (randomly shuffled across platforms) for adversarial/cycle objectives; optimization proceeds jointly so that per-sample fidelity and platform-level alignment are co-learned within the same step (Fig. 1). Both generators and discriminators use 1 × 1 convolutional layers that act as fully connected mappings across genes, enabling sample-wise mappings suitable for transcriptomics.

During the training process, we set the cycle-consistency weight (*λ*) to 10, as the default value defined by CycleGAN. We also set the newly introduced feedback loss weight (*β*) to 10. The choice of *λ* and *β* is further justified through a sensitivity analysis described in the Results section. The actual epoch number was determined by the training sample size. The other parameters, if not specified, used the default value from the original CycleGAN algorithm. During the training process, we rarely observed in some specific epoch the losses spiked to an abnormally high value, which would largely affect the training process. For such cases, we roll back the model checkpoints to address that issue to keep the model training process smooth. The threshold of loss increase to trigger rollback is ≥3 times the last observed losses of the training samples. Particularly, we used the sum of two feedback losses in this monitoring process.

#### Other implementation details and training configuration

Both generators (G1 and G2) were implemented as fully connected neural networks using 1 × 1 convolutional layers to emulate dense mappings across gene features. Each generator consisted of an encoder–decoder structure with multiple hidden layers and nonlinear activations (ReLU), followed by a linear output layer to match the target expression scale. Discriminators were implemented as multilayer perceptrons with fully connected layers and LeakyReLU activations, trained to distinguish real from generated profiles in each domain.

Models were trained using the Adam optimizer with standard parameters (learning rate = 0.0002, *β*1 = 0.5, *β*2 = 0.999). Training was performed using mini-batches consisting of both paired and unpaired samples at each iteration: one paired microarray–RNA-seq batch was used to compute the feedback loss, while a randomly shuffled unpaired batch was used for adversarial and cycle-consistency objectives. Batch size was set to 1, and training was conducted for up to 500 epochs, depending on dataset size, as described above.

To stabilize training, we applied a checkpoint rollback strategy when abrupt loss spikes were observed, defined as a ≥ 3-fold increase in the monitored loss relative to the previous iteration. Input gene expression values were log2-transformed (for RNA-seq) and standardized prior to training to ensure comparable scale across platforms. All other hyperparameters followed the default settings of the original CycleGAN framework unless otherwise specified. The full implementation of GANomics, including model architecture, training scripts, and example workflows for reproducing key results, is publicly available at https://github.com/seldas/GANomics.

### Demonstration datasets

The NB dataset was used in this study. The NB dataset is curated by the MAQC/SEQC consortium^32^, and has served as the benchmark dataset for many research^33,34^. It encompasses a total of 498 primary NB samples, each characterized by both microarray and RNA-seq data. The processed data for this dataset can be accessed through the GEO repository (GSE49710, GSE49711). To ensure the quality and compatibility of the data, as well as to conduct paired information between Microarray and RNA-seq gene expression profiles, a preprocessing step was undertaken. This step resulted in a refined dataset containing 10,042 genes that were consistently and uniquely (i.e., one-to-one) mapped between the Microarray and RNA-seq platforms. For detailed information on this preprocessing procedure, readers are referred to the study published elsewhere^32^. In this study, we investigated multiple training sample sizes. For each round, a subset of the dataset was utilized to develop a GANomics model, while the remaining samples were reserved as a testing set to evaluate model performance. For example, when 400 samples were used for training, the remaining 98 samples (i.e., 498–400) served as the testing set.

While primarily demonstrated by the NB dataset, we also validated GANomics on three other datasets, TCGA, NCI-60, and METSIM. First, we demonstrated GANomics on the TCGA benchmark datasets; the primary dataset we used from TCGA is the BRCA dataset, and its processed gene expression profiles are obtained via Synapse (microarray: syn2319914; RNA-seq: syn2320006, syn2320114). Note that multiple RNA-seq expression profiles were provided for the BRCA dataset, and we will analyze them all to see whether they can be transformed to/from Microarray. Similarly, we obtained transcriptomics profiles of two other TCGA datasets, LUSC and LAML. Both were also downloaded from Synapse. For LAML and LUSC, we only used the one RNA-seq profile, although they also provided two RNA-seq expression profiles, like BRCA.

The NCI-60 dataset is a panel of 60 diverse human cancer cell lines, developed by the U.S. National Cancer Institute, each with multiple profiles from different platforms. First, we focused on two profiles: Microarray with Affymetrix_HG_133_plus_2.0 with RMA normalization and RNA-seq with composite expression values. Raw expression profiles were downloaded from CellMiner^35^. Similarly, we also evaluated the translation from two other Microarray platforms (HG_U133(A-B) and HG_U95(A-E)) to RNA-seq in NCI60 datasets.

METSIM benchmark dataset was retrieved from GSE70353^36^ and GSE135134^37^ for its Microarray and RNA-seq data. Similarly, we processed the RNA-seq TPMs data with log2 transformation to make its value in range with the Microarray values.

For all datasets that use FPKM, log2 transformation is applied to make its value in the range of the Microarray values. From preliminary results, we observed that using the raw FPKM value will significantly increase the required training sample size as well as training epochs to develop a good GANomics model (Fig. S1).

In total, 9 benchmark datasets were analyzed in this study. The details of all benchmark datasets are summarized in Table 4.

**Table 4:** Benchmark dataset overview

Datasets	Microarray	RNA-seq	#Sample	#Mapped gene
NB	AG_020382 (GSE49710)	HiSeq (GSE49711)	498	10042
BRCA-1	AG_4502A (syn2319914)	HiSeq (syn2320006)	547	15697
BRCA-2	AG_4502A (syn2319914)	HiSeq (syn2320114)	588	15686
LAML	Affy_HG_U133_Plus2 (syn3591384)	GA (syn5824049)	172	17958
LUSC	AG_4502A (syn2320167)	HiSeq (syn2320155)	150	15889
NCI60-1	Affy_HG_U133_Plus2	Composite	60	8784
NCI60-2	Affy_HG_U133 A-B	Composite	60	7864
NCI60-3	Affy_HG_U95 A-E	Composite	60	6521
METSIM	Affy_HG_U219 (GSE70353)	HiSeq (GSE135134)	331	6801

### Related cross-platform translation algorithm

Beyond GANomics, we evaluated TDM^17^, QN^18^, ComBat^19^, YuGene^20^, and CuBlock^21^. For TDM and QN, we learn the target distribution from the training set of the destination platform and then transform held-out testing samples from the source to that reference (RNA-seq to Microarray and Microarray to RNA-seq). For ComBat, we fit parameters on training data and apply them to test; YuGene rescales each sample to the cumulative proportion scale; CuBlock performs block-wise monotone normalization based on gene clusters learned on source training data. All methods used identical training samples and were evaluated on the same held-out test sets to ensure comparability. For example, in the NB dataset, we used 10 samples to train the model translator and to predict the remaining 488 testing samples, which were then compared to GANomics-10 trained with the same training samples.

### Gene and pathway analysis

To assess how closely synthetic profiles recapitulate real experiments, we performed differential expression analysis within each platform (microarray and RNA-seq) using Welch’s two-sample *t* test (unequal variances). Per-gene *P* values were adjusted for multiple testing using the Benjamini–Hochberg procedure to obtain FDR *q* values. For a given FDR threshold *τ*, we defined the DEG set ${\rm{S}}\left({\rm{\tau }}\right)=[{\rm{g}}:{{\rm{q}}}_{g}\le {\rm{\tau }}]$. Concordance between real and synthetic DEGs was quantified with the Jaccard index (Eq. 6) across a range of FDR thresholds (*τ* ∈[10^−4^, 10^−3^, 10^−2^, 0.05, 0.1, 0.2, 0.5, 1.0]). Specifically, for each *τ*, we computed:

6 $${J}_{{DEG}}\left({\rm{\tau }}\right)=\frac{|{S}_{{real}}({\rm{\tau }})\cap {S}_{{syn}}({\rm{\tau }})|}{|{S}_{{real}}({\rm{\tau }})\cup {S}_{{syn}}({\rm{\tau }})|}$$

and reported ${J}_{{DEG}}\left({\rm{\tau }}\right)$ together with $|{S}_{{real}}({\rm{\tau }})|$ to indicate the number of discoveries at each FDR level.

To evaluate biological conservation beyond single genes, we performed two pathway-level enrichments on the full differential-expression results from real and synthetic data. First, we used the MSigDB Hallmark gene-set collection^38^. For each platform, genes were scored by an effect-size–based statistic (e.g., absolute Cohen’s *d* or |*t*|), and enrichment *P* values were computed per Hallmark set; multiple testing within each platform was controlled by Benjamini–Hochberg FDR, yielding pathway-level *q* values. We compared pathway rankings between real and synthetic results using (i) Spearman correlation of −log_10_(*q*) over shared Hallmark sets and (ii) overlap of the top K pathways (e.g., *K* = 10, 20, 50). To confirm that concordance exceeded chance, we conducted a size-matched permutation test that randomizes gene membership of Hallmark sets on the synthetic side (preserving each set’s size) and recomputed rank concordance to obtain a one-sided permutation *P* value. We additionally assessed pathway-rank stability via bootstrap resampling of samples (fraction 0.8, repeated B times), recomputing enrichment each time and summarizing the distribution of rank correlations and top *K* overlaps. Only Hallmark sets with at least five genes present in our expression universe were considered.

Second, we performed pathway enrichment analysis (PEA) based on both real and synthetic differentially expressed genes (DEGs) with DAVID^39^ to evaluate the enriched pathways from the DEG gene list with a *p* value threshold of 0.05.

### Developing predictive classifiers with real and synthetic gene expression profiles

To evaluate the predictive utility of synthetic profiles from GANomics, we implemented the RF algorithm to develop gene expression-based predictive models (i.e., classifiers, to differentiate them from the GANomics model) using both real and synthetic profiles and validated them on both profiles. The RF model, with default pre-set parameters (e.g., number of decision trees, *n* = 100), was evaluated using multiple statistical metrics, including recall, precision, specificity, F1-score, MCC, and predictive accuracy, to assess classification performance. This approach allowed for a direct comparison of predictive accuracy between real and synthetic datasets, providing insights into the reliability of synthetic profiles for downstream classification tasks.

### Combining strategies of using real and synthetic profiles in training classifier

To evaluate the impact of synthetic data on downstream prediction, we considered two strategies for combining real and GANomics-generated profiles. In the averaged approach, synthetic and real profiles from the same sample were combined by element-wise averaging, preserving sample identity while reducing noise. In the stacked approach, real and synthetic samples were treated as independent training instances, effectively doubling the training set size and enabling data augmentation.

## Supplementary information

GANomics Supplementary file

## Data Availability

All datasets analyzed in this study are publicly available. NB data are available at GEO under accession GSE49710 and GSE49711. TCGA datasets, including BRCA and LAML, are available in Synapse, as BRCA (microarray: syn2319914; RNA-seq: syn2320006, syn2320114), LUSC (microarray: syn2320167; RNA-seq: syn2320155) and LAML (microarray: syn3591384; RNA-seq: syn5824049). NCI-60 microarray and RNA-seq profiles are available via CellMiner (https://discover.nci.nih.gov/cellminer). METSIM microarray and RNA-seq data are available from GEO under accessions GSE70353 and GSE135134, respectively.
