# Supplementary material for: GANomics: bridging legacy and modern transcriptomic platforms for clinical applications
Source: NPJ Genom Med. 2026 Jun 19;11:56. doi: 10.1038/s41525-026-00585-w (PMC13642247; doi:10.1038/s41525-026-00585-w)
Supplement: Supplementary file 1 [file 41525_2026_585_MOESM1_ESM.pdf]

GANomics: Bridging Legacy and Modern Transcriptomic Platforms for Clinical Applications

Leihong Wu[1$*], Hadi Salman[1, 2$†], Weida Tong[1]

[1]Division of Bioinformatics and Biostatistics, National Center for Toxicological Research, FDA, Jefferson, 72079, Arkansas, USA.
[2] Department of Statistics, Texas A&M University, College Station, TX 77843, USA

[$] Contributed equally

[†] Dr. Hadi Salman contributed to this work while at the U.S. Food and Drug Administration (NCTR). His current affiliation is with Texas A&M University.

[*] Corresponding author

E-mails:
Leihong Wu: leihong.wu@fda.hhs.gov
Hadi Salman: hadi.salman@tamu.edu
Weida Tong: weida.tong@fda.hhs.gov

Supplementary Table and Figures

Table S1. *Detailed Performance of predictive classifiers developed with real and synthetic data, with different GANomics training sample size.*

Training Sample	Trained Epochs	Endpoint	Configuration	recall	precision	specificity	f1	mcc	accuracy
2	2000	D_FAV_All	C1 - Microarray	0.913	0.857	0.921	0.884	0.822	0.919
2	2000	D_FAV_All	C2 - Microarray	1.000	0.341	0.000	0.508	0.000	0.341
2	2000	D_FAV_All	C3 - Microarray	0.891	0.328	0.056	0.480	-0.095	0.341
2	2000	D_FAV_All	C4 - Microarray	0.848	0.951	0.978	0.897	0.851	0.933
2	2000	D_FAV_All	C1 - RNAseq	0.891	0.854	0.921	0.872	0.805	0.911
2	2000	D_FAV_All	C2 - RNAseq	0.000	0.000	1.000	0.000	0.000	0.659
2	2000	D_FAV_All	C3 - RNAseq	1.000	0.341	0.000	0.508	0.000	0.341
2	2000	D_FAV_All	C4 - RNAseq	0.783	0.947	0.978	0.857	0.801	0.911
5	2000	D_FAV_All	C1 - Microarray	0.935	0.860	0.921	0.896	0.840	0.926
5	2000	D_FAV_All	C2 - Microarray	0.130	1.000	1.000	0.231	0.300	0.704
5	2000	D_FAV_All	C3 - Microarray	0.913	0.618	0.708	0.737	0.589	0.778
5	2000	D_FAV_All	C4 - Microarray	0.913	0.857	0.921	0.884	0.822	0.919
5	2000	D_FAV_All	C1 - RNAseq	0.826	0.844	0.921	0.835	0.751	0.889
5	2000	D_FAV_All	C2 - RNAseq	0.196	1.000	1.000	0.327	0.372	0.726
5	2000	D_FAV_All	C3 - RNAseq	0.870	0.870	0.933	0.870	0.802	0.911
5	2000	D_FAV_All	C4 - RNAseq	0.891	0.774	0.865	0.828	0.734	0.874
10	2000	D_FAV_All	C1 - Microarray	0.911	0.854	0.920	0.882	0.819	0.917
10	2000	D_FAV_All	C2 - Microarray	0.311	0.875	0.977	0.459	0.419	0.752
10	2000	D_FAV_All	C3 - Microarray	0.911	0.732	0.830	0.812	0.710	0.857
10	2000	D_FAV_All	C4 - Microarray	0.933	0.824	0.898	0.875	0.809	0.910
10	2000	D_FAV_All	C1 - RNAseq	0.889	0.889	0.943	0.889	0.832	0.925
10	2000	D_FAV_All	C2 - RNAseq	0.200	1.000	1.000	0.333	0.377	0.729
10	2000	D_FAV_All	C3 - RNAseq	0.889	0.816	0.898	0.851	0.772	0.895
10	2000	D_FAV_All	C4 - RNAseq	0.800	0.878	0.943	0.837	0.761	0.895
20	2000	D_FAV_All	C1 - Microarray	0.909	0.851	0.915	0.879	0.812	0.913
20	2000	D_FAV_All	C2 - Microarray	0.364	1.000	1.000	0.533	0.521	0.778
20	2000	D_FAV_All	C3 - Microarray	0.955	0.545	0.573	0.694	0.516	0.706
20	2000	D_FAV_All	C4 - Microarray	0.955	0.955	0.976	0.955	0.930	0.968
20	2000	D_FAV_All	C1 - RNAseq	0.841	0.841	0.915	0.841	0.756	0.889
20	2000	D_FAV_All	C2 - RNAseq	0.455	0.952	0.988	0.615	0.566	0.802
20	2000	D_FAV_All	C3 - RNAseq	0.909	0.870	0.927	0.889	0.828	0.921
20	2000	D_FAV_All	C4 - RNAseq	0.955	0.857	0.915	0.903	0.850	0.929
30	400	D_FAV_All	C1 - Microarray	0.864	0.864	0.933	0.864	0.796	0.910
30	400	D_FAV_All	C2 - Microarray	0.523	1.000	1.000	0.687	0.650	0.842
30	400	D_FAV_All	C3 - Microarray	0.977	0.632	0.719	0.768	0.655	0.805
30	400	D_FAV_All	C4 - Microarray	0.909	0.870	0.933	0.889	0.833	0.925
30	400	D_FAV_All	C1 - RNAseq	0.841	0.860	0.933	0.851	0.778	0.902

30	400	D_FAV_All	C2 - RNAseq	0.568	1.000	1.000	0.725	0.684	0.857
30	400	D_FAV_All	C3 - RNAseq	0.977	0.581	0.652	0.729	0.596	0.759
30	400	D_FAV_All	C4 - RNAseq	0.932	0.911	0.955	0.921	0.882	0.947
40	400	D_FAV_All	C1 - Microarray	0.881	0.860	0.929	0.871	0.805	0.913
40	400	D_FAV_All	C2 - Microarray	0.786	0.846	0.929	0.815	0.728	0.881
40	400	D_FAV_All	C3 - Microarray	0.929	0.907	0.952	0.918	0.876	0.944
40	400	D_FAV_All	C4 - Microarray	0.881	0.841	0.917	0.860	0.789	0.905
40	400	D_FAV_All	C1 - RNAseq	0.857	0.837	0.917	0.847	0.769	0.897
40	400	D_FAV_All	C2 - RNAseq	0.762	0.970	0.988	0.853	0.804	0.913
40	400	D_FAV_All	C3 - RNAseq	1.000	0.689	0.774	0.816	0.730	0.849
40	400	D_FAV_All	C4 - RNAseq	0.857	0.878	0.940	0.867	0.803	0.913
**50**	400	D_FAV_All	C1 - Microarray	0.905	0.864	0.919	0.884	0.816	0.914
**50**	400	D_FAV_All	C2 - Microarray	0.857	0.973	0.986	0.911	0.870	0.940
**50**	400	D_FAV_All	C3 - Microarray	0.952	0.755	0.824	0.842	0.749	0.871
**50**	400	D_FAV_All	C4 - Microarray	0.905	0.905	0.946	0.905	0.851	0.931
**50**	400	D_FAV_All	C1 - RNAseq	0.857	0.857	0.919	0.857	0.776	0.897
**50**	400	D_FAV_All	C2 - RNAseq	0.857	0.923	0.959	0.889	0.831	0.922
**50**	400	D_FAV_All	C3 - RNAseq	0.952	0.851	0.905	0.899	0.840	0.922
**50**	400	D_FAV_All	C4 - RNAseq	0.976	0.872	0.919	0.921	0.876	0.940
100	200	D_FAV_All	C1 - Microarray	0.947	0.837	0.903	0.889	0.828	0.918
100	200	D_FAV_All	C2 - Microarray	0.868	0.892	0.944	0.880	0.818	0.918
100	200	D_FAV_All	C3 - Microarray	0.974	0.804	0.875	0.881	0.818	0.909
100	200	D_FAV_All	C4 - Microarray	0.974	0.881	0.931	0.925	0.885	0.945
100	200	D_FAV_All	C1 - RNAseq	0.868	0.805	0.889	0.835	0.745	0.882
100	200	D_FAV_All	C2 - RNAseq	0.895	0.810	0.889	0.850	0.767	0.891
100	200	D_FAV_All	C3 - RNAseq	0.974	0.881	0.931	0.925	0.885	0.945
100	200	D_FAV_All	C4 - RNAseq	0.974	0.881	0.931	0.925	0.885	0.945
150	200	D_FAV_All	C1 - Microarray	0.778	0.903	0.941	0.836	0.739	0.874
150	200	D_FAV_All	C2 - Microarray	0.778	0.903	0.941	0.836	0.739	0.874
150	200	D_FAV_All	C3 - Microarray	0.889	0.889	0.922	0.889	0.810	0.908
150	200	D_FAV_All	C4 - Microarray	0.917	0.846	0.882	0.880	0.791	0.897
150	200	D_FAV_All	C1 - RNAseq	0.861	0.861	0.902	0.861	0.763	0.885
150	200	D_FAV_All	C2 - RNAseq	0.778	0.903	0.941	0.836	0.739	0.874
150	200	D_FAV_All	C3 - RNAseq	0.972	0.795	0.824	0.875	0.784	0.885
150	200	D_FAV_All	C4 - RNAseq	0.944	0.829	0.863	0.883	0.796	0.897

Table S2. *Detailed performance of GANomics compared to other approaches, in other datasets*

	Pearson	Spearman	L1	L2	MAE	RMSE	Dataset
**GANomics (MA)**	0.86 (95% CI 0.85–0.86)	0.80 (95% CI 0.79–0.81)	7622.13 (95% CI 7479.89–7767.38)	86.97 (95% CI 85.46–88.53)	0.49 (95% CI 0.48–0.49)	0.69 (95% CI 0.68–0.71)	BRCA1
**ComBat (MA)**	0.15 (95% CI 0.15–0.16)	0.15 (95% CI 0.14–0.16)	47766.66 (95% CI 47629.22–47905.58)	456.14 (95% CI 455.08–457.21)	3.04 (95% CI 3.03–3.05)	3.64 (95% CI 3.63–3.65)	BRCA1
**YuGene (MA)**	0.27 (95% CI 0.27–0.28)	0.24 (95% CI 0.23–0.24)	16325.72 (95% CI 16231.31–16426.09)	175.50 (95% CI 174.55–176.45)	1.04 (95% CI 1.03–1.05)	1.40 (95% CI 1.39–1.41)	BRCA1
**CuBlock (MA)**	-0.02 (95% CI -0.02–-0.01)	0.84 (95% CI 0.83–0.85)	344444475.10 (95% CI 230042832.57–482593764.88)	328647046.38 (95% CI 214281184.49–466682899.19)	21943.33 (95% CI 14655.21–30744.33)	2623139.60 (95% CI 1710313.44–3724890.91)	BRCA1
**TDM (MA)**	0.14 (95% CI 0.13–0.14)	0.24 (95% CI 0.23–0.24)	146194.39 (95% CI 146107.20–146282.75)	1179.11 (95% CI 1178.44–1179.79)	9.31 (95% CI 9.31–9.32)	9.41 (95% CI 9.41–9.42)	BRCA1
**Quantile (MA)**	0.25 (95% CI 0.25–0.26)	0.24 (95% CI 0.23–0.24)	19894.14 (95% CI 19815.09–19978.05)	208.57 (95% CI 207.82–209.38)	1.27 (95% CI 1.26–1.27)	1.66 (95% CI 1.66–1.67)	BRCA1
**GANomics (RS)**	0.96 (95% CI 0.95–0.96)	0.96 (95% CI 0.95–0.96)	6496.67 (95% CI 6260.12–6845.65)	78.18 (95% CI 75.72–81.67)	0.41 (95% CI 0.40–0.44)	0.62 (95% CI 0.60–0.65)	BRCA1
**ComBat (RS)**	0.23 (95% CI 0.23–0.24)	0.21 (95% CI 0.20–0.21)	42497.44 (95% CI 42373.64–42619.77)	421.65 (95% CI 420.64–422.64)	2.71 (95% CI 2.70–2.72)	3.37 (95% CI 3.36–3.37)	BRCA1
**YuGene (RS)**	0.24 (95% CI 0.23–0.24)	0.24 (95% CI 0.23–0.24)	36034.97 (95% CI 35908.86–36160.65)	370.54 (95% CI 369.65–371.42)	2.30 (95% CI 2.29–2.30)	2.96 (95% CI 2.95–2.96)	BRCA1
**CuBlock (RS)**	-0.01 (95% CI -0.01–-0.01)	0.95 (95% CI 0.95–0.95)	272533469.13 (95% CI 112867613.51–529074470.68)	265717482.72 (95% CI 106133535.49–522380088.25)	17362.14 (95% CI 7190.39–33705.45)	2120859.02 (95% CI 847118.77–4169445.35)	BRCA1
**TDM (RS)**	0.25 (95% CI 0.24–0.25)	0.22 (95% CI 0.22–0.23)	89890.87 (95% CI 89696.00–90089.99)	816.14 (95% CI 814.91–817.38)	5.73 (95% CI 5.71–5.74)	6.51 (95% CI 6.50–6.52)	BRCA1
**Quantile (RS)**	0.23 (95% CI 0.22–0.23)	0.24 (95% CI 0.23–0.24)	32055.39 (95% CI 31945.23–32167.58)	324.14 (95% CI 323.25–325.07)	2.04 (95% CI 2.04–2.05)	2.59 (95% CI 2.58–2.59)	BRCA1
**Baseline (MA vs RS)**	0.28 (95% CI 0.27–0.28)	0.24 (95% CI 0.23–0.24)	44164.17 (95% CI 44034.73–44292.59)	426.83 (95% CI 425.82–427.85)	2.81 (95% CI 2.81–2.82)	3.41 (95% CI 3.40–3.41)	BRCA1
**GANomics (MA)**	0.81 (95% CI 0.80–0.81)	0.74 (95% CI 0.74–0.75)	8729.78 (95% CI 8589.57–8873.40)	100.08 (95% CI 98.50–101.70)	0.56 (95% CI 0.55–0.57)	0.80 (95% CI 0.79–0.81)	BRCA2
**ComBat (MA)**	0.27 (95% CI 0.26–0.27)	0.23 (95% CI 0.22–0.23)	106342.86 (95% CI 106241.43–106444.76)	958.57 (95% CI 957.88–959.29)	6.78 (95% CI 6.77–6.79)	7.65 (95% CI 7.65–7.66)	BRCA2
**YuGene (MA)**	0.28 (95% CI 0.28–0.29)	0.25 (95% CI 0.25–0.26)	16293.04 (95% CI 16204.41–16381.39)	175.32 (95% CI 174.43–176.20)	1.04 (95% CI 1.03–1.04)	1.40 (95% CI 1.39–1.41)	BRCA2
**CuBlock (MA)**	-0.02 (95% CI -0.02–-0.01)	0.84 (95% CI 0.84–0.85)	125732800.48 (95% CI 56297056.45–237785719.05)	117513288.06 (95% CI 48878206.97–229368943.17)	8015.61 (95% CI 3589.00–15159.10)	938276.57 (95% CI 390264.60–1831380.17)	BRCA2
**TDM (MA)**	0.16 (95% CI 0.16–0.16)	0.25 (95% CI 0.25–0.26)	157102.25 (95% CI 157016.40–157185.99)	1265.94 (95% CI 1265.26–1266.58)	10.02 (95% CI 10.01–10.02)	10.11 (95% CI 10.10–10.11)	BRCA2
**Quantile (MA)**	0.27 (95% CI 0.27–0.28)	0.25 (95% CI 0.25–0.26)	19645.82 (95% CI 19576.13–19715.08)	205.27 (95% CI 204.61–205.95)	1.25 (95% CI 1.25–1.26)	1.64 (95% CI 1.63–1.64)	BRCA2
**GANomics (RS)**	0.96 (95% CI 0.96–0.97)	0.96 (95% CI 0.96–0.96)	11674.73 (95% CI 11424.09–11937.59)	133.97 (95% CI 131.56–136.42)	0.74 (95% CI 0.73–0.76)	1.07 (95% CI 1.05–1.09)	BRCA2
**ComBat (RS)**	0.26 (95% CI 0.26–0.27)	0.28 (95% CI 0.27–0.28)	126170.18 (95% CI 126021.76–126316.74)	1108.44 (95% CI 1107.38–1109.50)	8.04 (95% CI 8.03–8.05)	8.85 (95% CI 8.84–8.86)	BRCA2
**YuGene (RS)**	0.24 (95% CI 0.24–0.25)	0.25 (95% CI 0.25–0.26)	106601.17 (95% CI 106405.67–106798.85)	961.90 (95% CI 960.90–962.90)	6.80 (95% CI 6.78–6.81)	7.68 (95% CI 7.67–7.69)	BRCA2

**CuBlock (RS)**	-0.01 (95% CI -0.01–-0.00)	0.95 (95% CI 0.95–0.96)	36817316.69 (95% CI 27484720.79–48494679.78)	30446554.29 (95% CI 21768278.84–41363212.96)	2347.15 (95% CI 1752.18–3091.59)	243098.37 (95% CI 173807.29–330261.66)	BRCA2
**TDM (RS)**	0.29 (95% CI 0.28–0.29)	0.25 (95% CI 0.25–0.26)	139662.07 (95% CI 139524.69–139803.01)	1221.95 (95% CI 1220.79–1223.14)	8.90 (95% CI 8.89–8.91)	9.76 (95% CI 9.75–9.77)	BRCA2
**Quantile (RS)**	0.25 (95% CI 0.25–0.25)	0.25 (95% CI 0.25–0.26)	56895.62 (95% CI 56745.20–57049.44)	578.39 (95% CI 577.11–579.72)	3.63 (95% CI 3.62–3.64)	4.62 (95% CI 4.61–4.63)	BRCA2
**Baseline (MA vs RS)**	0.29 (95% CI 0.28–0.29)	0.25 (95% CI 0.25–0.26)	113753.75 (95% CI 113592.25–113914.03)	1011.31 (95% CI 1010.34–1012.30)	7.25 (95% CI 7.24–7.26)	8.07 (95% CI 8.07–8.08)	BRCA2
**GANomics (MA)**	0.81 (95% CI 0.80–0.83)	0.75 (95% CI 0.73–0.77)	8529.29 (95% CI 8246.70–8838.94)	96.42 (95% CI 93.29–99.89)	0.54 (95% CI 0.52–0.56)	0.76 (95% CI 0.74–0.79)	LUSC
**ComBat (MA)**	0.21 (95% CI 0.20–0.22)	0.17 (95% CI 0.16–0.18)	45612.10 (95% CI 45359.21–45865.39)	430.79 (95% CI 428.68–432.85)	2.87 (95% CI 2.85–2.89)	3.42 (95% CI 3.40–3.43)	LUSC
**YuGene (MA)**	0.24 (95% CI 0.24–0.25)	0.20 (95% CI 0.19–0.21)	16089.76 (95% CI 15915.93–16270.75)	171.16 (95% CI 169.36–172.97)	1.01 (95% CI 1.00–1.02)	1.36 (95% CI 1.34–1.37)	LUSC
**CuBlock (MA)**	-0.01 (95% CI -0.01–-0.01)	0.84 (95% CI 0.83–0.85)	326025336873.79 (95% CI 26156796579.08–795633183272.98)	325116164979.39 (95% CI 25604776442.26–794492841322.02)	20518933.66 (95% CI 1646220.44–50074465.56)	2579231243.07 (95% CI 203129362.63–6302918708.64)	LUSC
**TDM (MA)**	0.13 (95% CI 0.13–0.14)	0.20 (95% CI 0.19–0.21)	136132.14 (95% CI 135906.98–136343.30)	1092.63 (95% CI 1090.78–1094.39)	8.57 (95% CI 8.55–8.58)	8.67 (95% CI 8.65–8.68)	LUSC
**Quantile (MA)**	0.23 (95% CI 0.23–0.24)	0.20 (95% CI 0.19–0.21)	19835.94 (95% CI 19700.73–19973.43)	207.51 (95% CI 206.17–208.85)	1.25 (95% CI 1.24–1.26)	1.65 (95% CI 1.64–1.66)	LUSC
**GANomics (RS)**	0.95 (95% CI 0.94–0.95)	0.95 (95% CI 0.94–0.95)	7684.10 (95% CI 7332.32–8078.06)	90.41 (95% CI 86.69–94.57)	0.48 (95% CI 0.46–0.51)	0.72 (95% CI 0.69–0.75)	LUSC
**ComBat (RS)**	0.15 (95% CI 0.14–0.16)	0.09 (95% CI 0.08–0.10)	43499.53 (95% CI 43250.71–43743.91)	435.46 (95% CI 433.19–437.65)	2.74 (95% CI 2.72–2.75)	3.45 (95% CI 3.44–3.47)	LUSC
**YuGene (RS)**	0.22 (95% CI 0.21–0.23)	0.20 (95% CI 0.19–0.21)	36247.98 (95% CI 36028.74–36463.51)	373.11 (95% CI 371.45–374.73)	2.28 (95% CI 2.27–2.29)	2.96 (95% CI 2.95–2.97)	LUSC
**CuBlock (RS)**	-0.00 (95% CI -0.01–-0.00)	0.95 (95% CI 0.94–0.95)	147897918278.45 (95% CI 52102144536.87–274129845604.51)	143074706453.53 (95% CI 48524244457.73–268236967449.52)	9308195.50 (95% CI 3279133.02–17252806.70)	1135048923.21 (95% CI 384955473.87–2127993749.66)	LUSC
**TDM (RS)**	0.22 (95% CI 0.21–0.23)	0.19 (95% CI 0.18–0.20)	93667.29 (95% CI 93231.49–94072.81)	842.15 (95% CI 839.47–844.72)	5.90 (95% CI 5.87–5.92)	6.68 (95% CI 6.66–6.70)	LUSC
**Quantile (RS)**	0.22 (95% CI 0.21–0.22)	0.20 (95% CI 0.19–0.21)	33165.44 (95% CI 32956.56–33375.17)	330.59 (95% CI 328.92–332.25)	2.09 (95% CI 2.07–2.10)	2.62 (95% CI 2.61–2.64)	LUSC
**Baseline (MA vs RS)**	0.26 (95% CI 0.25–0.26)	0.20 (95% CI 0.19–0.21)	43822.92 (95% CI 43587.22–44054.93)	425.04 (95% CI 422.98–427.04)	2.76 (95% CI 2.74–2.77)	3.37 (95% CI 3.36–3.39)	LUSC
**GANomics (MA)**	0.93 (95% CI 0.93–0.94)	0.94 (95% CI 0.94–0.95)	12621.64 (95% CI 12349.30–12894.82)	133.23 (95% CI 130.90–135.48)	0.70 (95% CI 0.69–0.72)	0.99 (95% CI 0.98–1.01)	LAML
**ComBat (MA)**	0.73 (95% CI 0.73–0.74)	0.84 (95% CI 0.84–0.84)	197096.16 (95% CI 196698.19–197496.19)	1596.07 (95% CI 1592.60–1599.55)	10.98 (95% CI 10.95–11.00)	11.91 (95% CI 11.88–11.94)	LAML
**YuGene (MA)**	0.84 (95% CI 0.83–0.84)	0.86 (95% CI 0.86–0.86)	167670.09 (95% CI 167271.88–168045.34)	1295.84 (95% CI 1293.72–1297.85)	9.34 (95% CI 9.31–9.36)	9.67 (95% CI 9.65–9.68)	LAML
**CuBlock (MA)**	-0.00 (95% CI -0.00–-0.00)	0.92 (95% CI 0.91–0.92)	7696696315128.96 (95% CI 313427906577.24–20129430105827.82)	7690333478885.21 (95% CI 309719163047.48–20120766252037.62)	428594293.08 (95% CI 17453386.04–1120917145.89)	57387352440.19 (95% CI 2311208326.15–150146610344.37)	LAML
**TDM (MA)**	0.85 (95% CI 0.84–0.85)	0.86 (95% CI 0.86–0.86)	20728.70 (95% CI 20494.37–20957.28)	213.71 (95% CI 211.51–215.86)	1.15 (95% CI 1.14–1.17)	1.59 (95% CI 1.58–1.61)	LAML
**Quantile (MA)**	0.82 (95% CI 0.82–0.83)	0.86 (95% CI 0.86–0.86)	21616.35 (95% CI 21402.67–21819.43)	219.44 (95% CI 217.82–221.04)	1.20 (95% CI 1.19–1.22)	1.64 (95% CI 1.63–1.65)	LAML

**GANomics (RS)**	0.82 (95% CI 0.82–0.83)	0.87 (95% CI 0.86–0.87)	15039.02 (95% CI 14928.52–15155.90)	184.75 (95% CI 184.07–185.48)	0.84 (95% CI 0.83–0.84)	1.38 (95% CI 1.37–1.38)	LAML
**ComBat (RS)**	0.89 (95% CI 0.89–0.90)	0.90 (95% CI 0.90–0.90)	111354.30 (95% CI 111158.12–111554.10)	864.59 (95% CI 863.13–866.07)	6.20 (95% CI 6.19–6.21)	6.45 (95% CI 6.44–6.46)	LAML
**YuGene (RS)**	0.86 (95% CI 0.86–0.86)	0.86 (95% CI 0.86–0.86)	36557.86 (95% CI 36367.49–36745.10)	359.98 (95% CI 358.96–361.02)	2.04 (95% CI 2.03–2.05)	2.69 (95% CI 2.68–2.69)	LAML
**CuBlock (RS)**	-0.00 (95% CI -0.00–-0.00)	0.94 (95% CI 0.94–0.95)	12398797357870.35 (95% CI 1442657603241.69–28150597326320.36)	12386722899403.18 (95% CI 1437733061940.82–28138306179579.07)	690433085.97 (95% CI 80335093.18–1567579759.79)	92433082981.22 (95% CI 10728753722.72–209975665974.84)	LAML
**TDM (RS)**	0.88 (95% CI 0.87–0.88)	0.86 (95% CI 0.86–0.87)	12940.60 (95% CI 12738.98–13137.67)	140.70 (95% CI 138.96–142.42)	0.72 (95% CI 0.71–0.73)	1.05 (95% CI 1.04–1.06)	LAML
**Quantile (RS)**	0.88 (95% CI 0.88–0.89)	0.86 (95% CI 0.86–0.86)	12539.92 (95% CI 12345.90–12729.23)	135.62 (95% CI 133.89–137.33)	0.70 (95% CI 0.69–0.71)	1.01 (95% CI 1.00–1.02)	LAML
**Baseline (MA vs RS)**	0.85 (95% CI 0.84–0.85)	0.86 (95% CI 0.86–0.86)	132621.89 (95% CI 132257.75–132991.08)	1009.34 (95% CI 1006.82–1011.88)	7.39 (95% CI 7.36–7.41)	7.53 (95% CI 7.51–7.55)	LAML
**GANomics (MA)**	0.96 (95% CI 0.95–0.96)	0.96 (95% CI 0.96–0.97)	6297.54 (95% CI 4364.91–8359.35)	88.18 (95% CI 68.65–108.97)	0.72 (95% CI 0.50–0.95)	0.94 (95% CI 0.73–1.16)	NCI60-1
**ComBat (MA)**	0.75 (95% CI 0.72–0.78)	0.70 (95% CI 0.67–0.73)	40674.62 (95% CI 39349.57–41840.21)	461.89 (95% CI 447.80–474.22)	4.63 (95% CI 4.48–4.76)	4.93 (95% CI 4.78–5.06)	NCI60-1
**YuGene (MA)**	0.80 (95% CI 0.79–0.80)	0.78 (95% CI 0.78–0.79)	43761.09 (95% CI 43556.22–43959.22)	502.83 (95% CI 500.77–504.83)	4.98 (95% CI 4.96–5.00)	5.37 (95% CI 5.34–5.39)	NCI60-1
**CuBlock (MA)**	-0.00 (95% CI -0.01–0.00)	0.90 (95% CI 0.81–0.96)	151832547134.31 (95% CI 18375403796.68–332447239305.66)	118696740427.20 (95% CI 18053287932.33–254355133221.08)	17285126.04 (95% CI 2091917.55–37846907.94)	1266463358.54 (95% CI 192623888.28–2713903137.71)	NCI60-1
**TDM (MA)**	0.84 (95% CI 0.82–0.85)	0.78 (95% CI 0.78–0.79)	26147.14 (95% CI 24766.01–27658.34)	301.04 (95% CI 287.47–315.83)	2.98 (95% CI 2.82–3.15)	3.21 (95% CI 3.07–3.37)	NCI60-1
**Quantile (MA)**	0.83 (95% CI 0.82–0.84)	0.77 (95% CI 0.75–0.78)	8707.73 (95% CI 8421.06–9015.98)	120.65 (95% CI 116.68–124.74)	0.99 (95% CI 0.96–1.03)	1.29 (95% CI 1.24–1.33)	NCI60-1
**GANomics (RS)**	0.92 (95% CI 0.91–0.94)	0.88 (95% CI 0.86–0.90)	3857.24 (95% CI 3090.64–4786.54)	68.99 (95% CI 57.20–84.06)	0.44 (95% CI 0.35–0.54)	0.74 (95% CI 0.61–0.90)	NCI60-1
**ComBat (RS)**	0.81 (95% CI 0.80–0.82)	0.79 (95% CI 0.78–0.80)	38700.37 (95% CI 37241.44–39971.50)	443.95 (95% CI 426.51–459.23)	4.41 (95% CI 4.24–4.55)	4.74 (95% CI 4.55–4.90)	NCI60-1
**YuGene (RS)**	0.74 (95% CI 0.72–0.76)	0.78 (95% CI 0.78–0.79)	8758.39 (95% CI 7518.16–10214.08)	155.75 (95% CI 135.87–179.07)	1.00 (95% CI 0.86–1.16)	1.66 (95% CI 1.45–1.91)	NCI60-1
**CuBlock (RS)**	-0.01 (95% CI -0.02–-0.00)	0.87 (95% CI 0.85–0.89)	695840447861.17 (95% CI 248155114.37–2067173978469.25)	695605111109.79 (95% CI 199501916.94–2066934118941.05)	79216808.73 (95% CI 28250.81–235334013.94)	7421925674.32 (95% CI 2128633.58–22053649634.67)	NCI60-1
**TDM (RS)**	0.62 (95% CI 0.60–0.63)	0.78 (95% CI 0.78–0.79)	10946.14 (95% CI 9722.01–12396.10)	194.15 (95% CI 173.37–218.51)	1.25 (95% CI 1.11–1.41)	2.07 (95% CI 1.85–2.33)	NCI60-1
**Quantile (RS)**	0.86 (95% CI 0.84–0.87)	0.78 (95% CI 0.78–0.79)	5087.65 (95% CI 4650.53–5578.99)	89.23 (95% CI 82.75–97.03)	0.58 (95% CI 0.53–0.64)	0.95 (95% CI 0.88–1.04)	NCI60-1
**Baseline (MA vs RS)**	0.83 (95% CI 0.81–0.84)	0.78 (95% CI 0.78–0.79)	37186.50 (95% CI 35683.39–38487.36)	413.51 (95% CI 396.47–428.28)	4.23 (95% CI 4.06–4.38)	4.41 (95% CI 4.23–4.57)	NCI60-1
**GANomics (MA)**	0.96 (95% CI 0.95–0.96)	0.95 (95% CI 0.95–0.96)	4409.92 (95% CI 3216.52–6084.08)	66.03 (95% CI 53.23–83.85)	0.56 (95% CI 0.41–0.77)	0.74 (95% CI 0.60–0.95)	NCI60-2
**ComBat (MA)**	0.75 (95% CI 0.74–0.75)	0.74 (95% CI 0.73–0.74)	33058.39 (95% CI 32693.30–33487.04)	394.38 (95% CI 390.73–398.42)	4.20 (95% CI 4.16–4.26)	4.45 (95% CI 4.41–4.49)	NCI60-2
**YuGene (MA)**	0.73 (95% CI 0.72–0.75)	0.73 (95% CI 0.71–0.74)	44517.51 (95% CI 44468.13–44563.65)	521.97 (95% CI 521.10–522.92)	5.66 (95% CI 5.65–5.67)	5.89 (95% CI 5.88–5.90)	NCI60-2

**CuBlock (MA)**	-0.01 (95% CI -0.01–0.00)	0.94 (95% CI 0.92–0.96)	3914770650.10 (95% CI 258781828.90–10683489858.12)	3840633510.33 (95% CI 228818523.72–10603854559.14)	497809.09 (95% CI 32907.15–1358531.26)	43309295.03 (95% CI 2580295.39–119575446.16)	NCI60-2
**TDM (MA)**	0.79 (95% CI 0.78–0.80)	0.73 (95% CI 0.71–0.74)	7258.69 (95% CI 7045.35–7479.13)	105.51 (95% CI 102.69–108.47)	0.92 (95% CI 0.90–0.95)	1.19 (95% CI 1.16–1.22)	NCI60-2
**Quantile (MA)**	0.77 (95% CI 0.75–0.78)	0.72 (95% CI 0.71–0.74)	7479.36 (95% CI 7320.50–7700.98)	109.05 (95% CI 107.11–111.56)	0.95 (95% CI 0.93–0.98)	1.23 (95% CI 1.21–1.26)	NCI60-2
**GANomics (RS)**	0.90 (95% CI 0.88–0.92)	0.89 (95% CI 0.87–0.91)	3809.62 (95% CI 3421.43–4281.47)	68.29 (95% CI 62.40–76.12)	0.48 (95% CI 0.44–0.54)	0.77 (95% CI 0.70–0.86)	NCI60-2
**ComBat (RS)**	0.73 (95% CI 0.71–0.75)	0.73 (95% CI 0.71–0.74)	44812.25 (95% CI 44210.00–45545.08)	529.76 (95% CI 522.46–538.69)	5.70 (95% CI 5.62–5.79)	5.97 (95% CI 5.89–6.07)	NCI60-2
**YuGene (RS)**	0.72 (95% CI 0.71–0.74)	0.73 (95% CI 0.71–0.74)	9431.02 (95% CI 8601.47–10066.20)	161.06 (95% CI 148.72–170.67)	1.20 (95% CI 1.09–1.28)	1.82 (95% CI 1.68–1.92)	NCI60-2
**CuBlock (RS)**	-0.02 (95% CI -0.02–-0.01)	0.91 (95% CI 0.89–0.92)	327685000.09 (95% CI 29349314.86–856223828.02)	301958810.47 (95% CI 21921504.82–800509968.90)	41669.00 (95% CI 3732.11–108878.92)	3405069.29 (95% CI 247200.08–9027032.21)	NCI60-2
**TDM (RS)**	0.82 (95% CI 0.80–0.83)	0.73 (95% CI 0.71–0.74)	5869.86 (95% CI 5629.45–6125.11)	91.68 (95% CI 88.43–95.16)	0.75 (95% CI 0.72–0.78)	1.03 (95% CI 1.00–1.07)	NCI60-2
**Quantile (RS)**	0.83 (95% CI 0.81–0.84)	0.73 (95% CI 0.71–0.74)	5727.32 (95% CI 5509.29–5972.98)	91.33 (95% CI 88.25–94.87)	0.73 (95% CI 0.70–0.76)	1.03 (95% CI 1.00–1.07)	NCI60-2
**Baseline (MA vs RS)**	0.79 (95% CI 0.78–0.80)	0.73 (95% CI 0.71–0.74)	36485.24 (95% CI 35887.42–37208.05)	423.56 (95% CI 416.88–431.52)	4.64 (95% CI 4.56–4.73)	4.78 (95% CI 4.70–4.87)	NCI60-2
**GANomics (MA)**	0.97 (95% CI 0.96–0.98)	0.97 (95% CI 0.96–0.98)	3404.78 (95% CI 2598.32–4270.42)	54.09 (95% CI 44.85–64.01)	0.52 (95% CI 0.40–0.65)	0.67 (95% CI 0.56–0.79)	NCI60-3
**ComBat (MA)**	0.74 (95% CI 0.73–0.75)	0.73 (95% CI 0.71–0.74)	19676.22 (95% CI 19367.68–19988.67)	269.63 (95% CI 266.61–272.62)	3.02 (95% CI 2.97–3.07)	3.34 (95% CI 3.30–3.38)	NCI60-3
**YuGene (MA)**	0.66 (95% CI 0.64–0.68)	0.64 (95% CI 0.62–0.66)	31575.50 (95% CI 31454.14–31648.06)	416.65 (95% CI 414.59–417.86)	4.84 (95% CI 4.82–4.85)	5.16 (95% CI 5.13–5.17)	NCI60-3
**CuBlock (MA)**	-0.01 (95% CI -0.02–0.00)	0.91 (95% CI 0.85–0.96)	492715706785.27 (95% CI 21834861226.41–1301816925385.00)	398339268004.27 (95% CI 18901650485.39–1028580173806.51)	75558304.98 (95% CI 3348391.54–199634553.81)	4932828548.64 (95% CI 234068314.68–12737407665.93)	NCI60-3
**TDM (MA)**	0.71 (95% CI 0.70–0.73)	0.64 (95% CI 0.62–0.66)	8656.80 (95% CI 8186.89–9091.31)	134.40 (95% CI 127.49–140.75)	1.33 (95% CI 1.26–1.39)	1.66 (95% CI 1.58–1.74)	NCI60-3
**Quantile (MA)**	0.70 (95% CI 0.68–0.72)	0.64 (95% CI 0.62–0.66)	7483.62 (95% CI 7287.09–7687.12)	122.37 (95% CI 119.63–125.09)	1.15 (95% CI 1.12–1.18)	1.52 (95% CI 1.48–1.55)	NCI60-3
**GANomics (RS)**	0.92 (95% CI 0.90–0.93)	0.90 (95% CI 0.88–0.91)	3301.62 (95% CI 3044.36–3594.33)	61.74 (95% CI 58.46–65.15)	0.51 (95% CI 0.47–0.55)	0.76 (95% CI 0.72–0.81)	NCI60-3
**ComBat (RS)**	0.60 (95% CI 0.57–0.62)	0.61 (95% CI 0.59–0.63)	31351.83 (95% CI 30735.33–32002.93)	430.49 (95% CI 422.57–439.16)	4.81 (95% CI 4.71–4.91)	5.33 (95% CI 5.23–5.44)	NCI60-3
**YuGene (RS)**	0.65 (95% CI 0.63–0.67)	0.64 (95% CI 0.62–0.66)	9691.42 (95% CI 8834.50–10457.67)	169.26 (95% CI 156.54–180.40)	1.49 (95% CI 1.35–1.60)	2.10 (95% CI 1.94–2.23)	NCI60-3
**CuBlock (RS)**	-0.01 (95% CI -0.02–-0.00)	0.91 (95% CI 0.88–0.93)	3697746091.42 (95% CI 289362429.64–8817437092.35)	3632658694.04 (95% CI 219609283.68–8730826713.62)	567052.00 (95% CI 44373.93–1352160.27)	44984976.26 (95% CI 2719528.38–108118065.99)	NCI60-3
**TDM (RS)**	0.74 (95% CI 0.72–0.75)	0.64 (95% CI 0.62–0.66)	6043.61 (95% CI 5657.18–6399.44)	102.39 (95% CI 95.81–108.15)	0.93 (95% CI 0.87–0.98)	1.27 (95% CI 1.19–1.34)	NCI60-3
**Quantile (RS)**	0.74 (95% CI 0.73–0.76)	0.64 (95% CI 0.62–0.66)	6287.76 (95% CI 6081.38–6484.21)	107.48 (95% CI 103.53–111.01)	0.96 (95% CI 0.93–0.99)	1.33 (95% CI 1.28–1.37)	NCI60-3
**Baseline (MA vs RS)**	0.72 (95% CI 0.70–0.73)	0.64 (95% CI 0.62–0.66)	22945.23 (95% CI 22355.49–23575.09)	305.05 (95% CI 298.65–311.93)	3.52 (95% CI 3.43–3.62)	3.78 (95% CI 3.70–3.86)	NCI60-3

**GANomics (MA)**	0.99 (95% CI 0.99–0.99)	0.96 (95% CI 0.96–0.96)	1663.53 (95% CI 1624.66–1703.41)	27.98 (95% CI 27.32–28.66)	0.24 (95% CI 0.24–0.25)	0.34 (95% CI 0.33–0.35)	METSIM
**ComBat (MA)**	0.04 (95% CI 0.04–0.04)	0.16 (95% CI 0.16–0.16)	176272.81 (95% CI 175592.42–176938.23)	17494.01 (95% CI 17404.82–17581.99)	25.92 (95% CI 25.82–26.02)	212.13 (95% CI 211.05–213.20)	METSIM
**YuGene (MA)**	0.19 (95% CI 0.19–0.19)	0.21 (95% CI 0.21–0.22)	28206.12 (95% CI 28190.69–28221.37)	385.78 (95% CI 385.52–386.04)	4.15 (95% CI 4.15–4.15)	4.68 (95% CI 4.67–4.68)	METSIM
**CuBlock (MA)**	0.01 (95% CI 0.00–0.02)	0.92 (95% CI 0.91–0.93)	32415282830748.02 (95% CI 6737404807065.90–76190539210793.08)	32407074512701.66 (95% CI 6732945146965.49–76184430649160.41)	4766252437.99 (95% CI 990649140.87–11202843583.41)	392964610232.44 (95% CI 81642950040.33–923804001008.91)	METSIM
**TDM (MA)**	0.16 (95% CI 0.16–0.16)	0.21 (95% CI 0.21–0.21)	21581.79 (95% CI 21535.42–21627.79)	352.62 (95% CI 351.82–353.40)	3.17 (95% CI 3.17–3.18)	4.28 (95% CI 4.27–4.29)	METSIM
**Quantile (MA)**	0.21 (95% CI 0.21–0.21)	0.22 (95% CI 0.22–0.22)	13619.99 (95% CI 13607.86–13631.94)	228.47 (95% CI 228.30–228.63)	2.00 (95% CI 2.00–2.00)	2.77 (95% CI 2.77–2.77)	METSIM
**GANomics (RS)**	0.98 (95% CI 0.98–0.98)	0.96 (95% CI 0.96–0.96)	1974.69 (95% CI 1899.83–2053.76)	36.55 (95% CI 35.23–37.98)	0.29 (95% CI 0.28–0.30)	0.44 (95% CI 0.43–0.46)	METSIM
**ComBat (RS)**	-0.32 (95% CI -0.32–-0.32)	-0.56 (95% CI -0.56–-0.56)	671654.49 (95% CI 671600.10–671706.56)	76319.37 (95% CI 76319.00–76319.75)	98.76 (95% CI 98.75–98.77)	925.44 (95% CI 925.44–925.44)	METSIM
**YuGene (RS)**	0.22 (95% CI 0.22–0.22)	0.21 (95% CI 0.21–0.22)	13418.54 (95% CI 13345.13–13494.05)	238.84 (95% CI 238.06–239.64)	1.97 (95% CI 1.96–1.98)	2.90 (95% CI 2.89–2.91)	METSIM
**CuBlock (RS)**	-0.00 (95% CI -0.00–-0.00)	0.94 (95% CI 0.94–0.94)	525236935762.26 (95% CI 96920197011.25–1091491566263.49)	524131689689.59 (95% CI 96190626385.18–1089960096410.95)	77229368.59 (95% CI 14250874.43–160489864.18)	6355563044.38 (95% CI 1166396923.32–13216735879.30)	METSIM
**TDM (RS)**	NA	NA	NA	NA	NA	NA	METSIM
**Quantile (RS)**	0.01 (95% CI 0.01–0.01)	0.21 (95% CI 0.21–0.22)	275033.16 (95% CI 275022.79–275043.97)	31200.65 (95% CI 31200.52–31200.78)	40.44 (95% CI 40.44–40.44)	378.34 (95% CI 378.33–378.34)	METSIM
**Baseline (MA vs RS)**	0.21 (95% CI 0.21–0.21)	0.21 (95% CI 0.21–0.22)	21525.56 (95% CI 21452.89–21600.81)	316.59 (95% CI 315.80–317.40)	3.17 (95% CI 3.15–3.18)	3.84 (95% CI 3.83–3.85)	METSIM

Table S3. *Detailed performance of Combined (stack or average pooling) data using GANomics-50*

Platform	Train_Size_Base	Scenario	Mean_Baseline	Mean_Scenario	Mean_Diff	p_ttest	sig_ttest	p_wilcoxon	sig_wilcoxon
MA	10	Real+Syn(avg)->Real	0.361	0.513	0.151	6.70E-10	***	1.75E-08	***
MA	10	Real+Syn(stack)->Real	0.361	0.646	0.285	2.37E-16	***	1.78E-15	***
MA	10	Syn->Real	0.361	0.491	0.130	5.03E-06	***	7.95E-06	***
MA	10	Syn->Syn	0.361	0.570	0.208	1.96E-12	***	3.78E-09	***
MA	20	Real+Syn(avg)->Real	0.713	0.746	0.033	1.74E-03	**	1.28E-03	**
MA	20	Real+Syn(stack)->Real	0.713	0.770	0.057	1.06E-06	***	2.87E-06	***
MA	20	Syn->Real	0.713	0.718	0.006	7.39E-01	ns	8.04E-01	ns
MA	20	Syn->Syn	0.713	0.770	0.057	1.29E-05	***	2.83E-05	***
MA	50	Real+Syn(avg)->Real	0.807	0.844	0.037	6.20E-07	***	7.77E-06	***
MA	50	Real+Syn(stack)->Real	0.807	0.841	0.035	9.10E-08	***	2.14E-06	***
MA	50	Syn->Real	0.807	0.819	0.013	1.55E-01	ns	1.29E-01	ns
MA	50	Syn->Syn	0.807	0.860	0.053	1.30E-08	***	3.44E-07	***
MA	100	Real+Syn(avg)->Real	0.848	0.872	0.024	1.16E-05	***	7.86E-05	***
MA	100	Real+Syn(stack)->Real	0.848	0.865	0.018	2.89E-03	**	4.88E-03	**
MA	100	Syn->Real	0.848	0.841	-0.007	3.43E-01	ns	3.18E-01	ns
MA	100	Syn->Syn	0.848	0.886	0.038	1.12E-08	***	1.88E-07	***
RS	10	Real+Syn(avg)->Real	0.421	0.544	0.122	4.03E-09	***	1.81E-08	***
RS	10	Real+Syn(stack)->Real	0.421	0.655	0.233	1.66E-14	***	5.33E-15	***
RS	10	Syn->Real	0.421	0.486	0.065	2.23E-03	**	3.40E-03	**
RS	10	Syn->Syn	0.421	0.583	0.161	4.14E-10	***	1.25E-08	***
RS	20	Real+Syn(avg)->Real	0.713	0.770	0.057	4.28E-09	***	1.99E-07	***
RS	20	Real+Syn(stack)->Real	0.713	0.778	0.065	1.34E-07	***	7.59E-08	***
RS	20	Syn->Real	0.713	0.753	0.040	2.73E-04	***	2.62E-04	***
RS	20	Syn->Syn	0.713	0.787	0.074	1.43E-08	***	4.46E-07	***
RS	50	Real+Syn(avg)->Real	0.815	0.850	0.035	2.71E-06	***	2.17E-05	***
RS	50	Real+Syn(stack)->Real	0.815	0.853	0.038	1.04E-07	***	1.67E-06	***
RS	50	Syn->Real	0.815	0.839	0.024	1.49E-02	*	7.13E-03	**
RS	50	Syn->Syn	0.815	0.867	0.052	1.14E-08	***	6.90E-07	***
RS	100	Real+Syn(avg)->Real	0.851	0.885	0.034	4.79E-06	***	2.41E-05	***
RS	100	Real+Syn(stack)->Real	0.851	0.877	0.027	8.30E-05	***	2.72E-04	***
RS	100	Syn->Real	0.851	0.860	0.009	3.34E-01	ns	4.49E-01	ns
RS	100	Syn->Syn	0.851	0.897	0.046	9.16E-09	***	2.44E-07	***

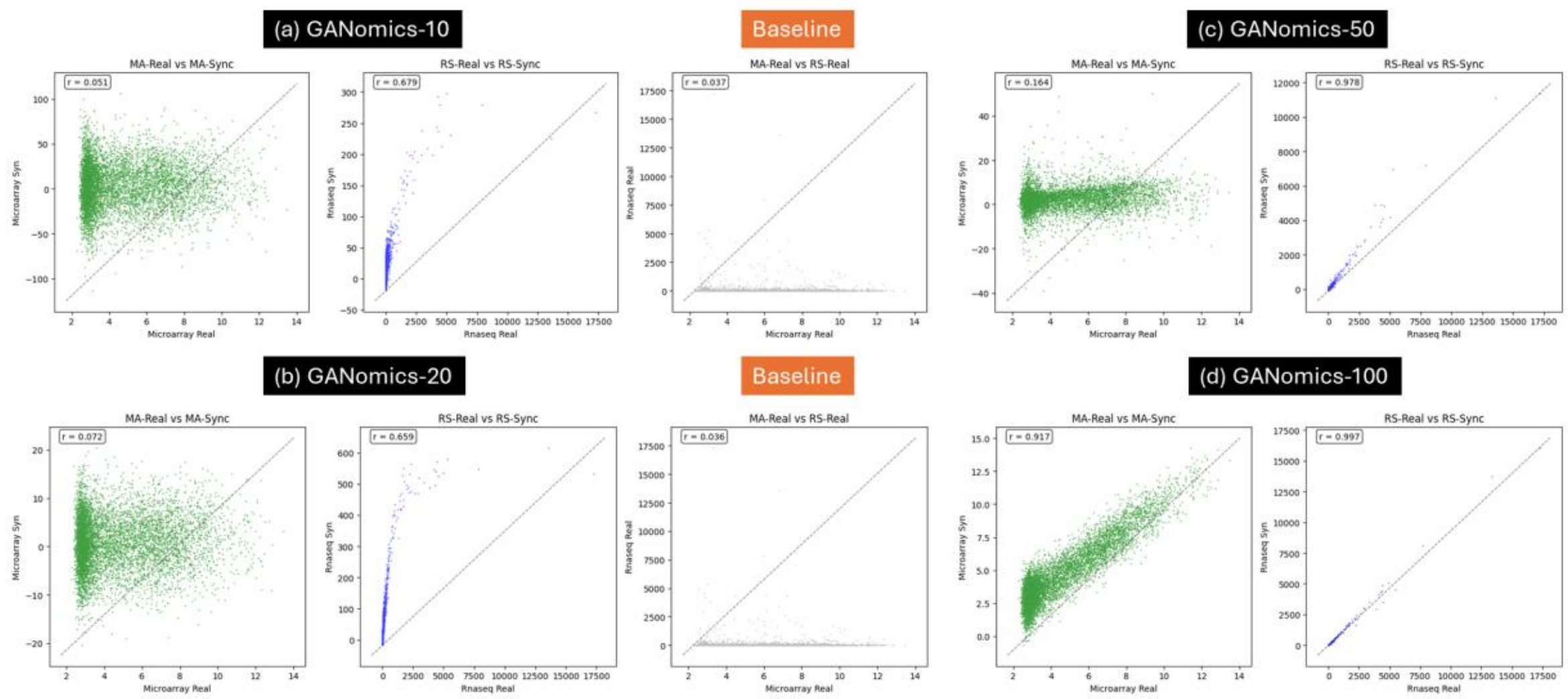

Figure S1. *Training GANomics with non-normalized transformed expression profiles with METSIM. RNA-seq data was not log2 transformed in this analysis, which resulted in a large increase of training sample needed. As shown in the figure, GANomics-10 did not work well, while using 100 samples for training (GANomics-100) showed in a more acceptable translation result.*

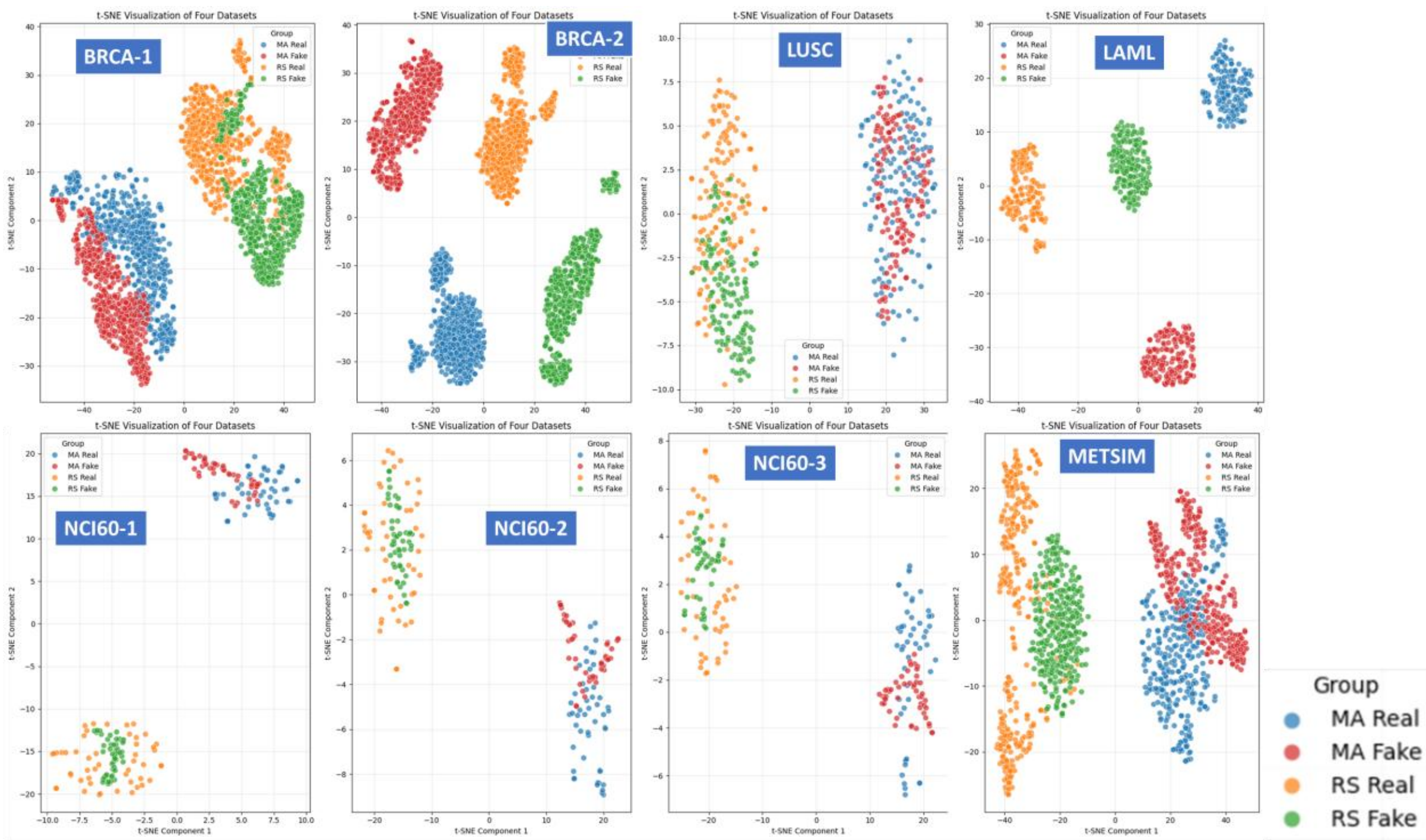

Figure S2. *t-SNE analysis of real and synthetic expression profiles in testing samples for BRCA, NCI60 and METSIM datasets, using GANomics-10. Samples are visualized in different color by its category; blue: Microarray real; red: Microarray synthetic; yellow: RNA-seq real; green: RNA-seq synthetic.*

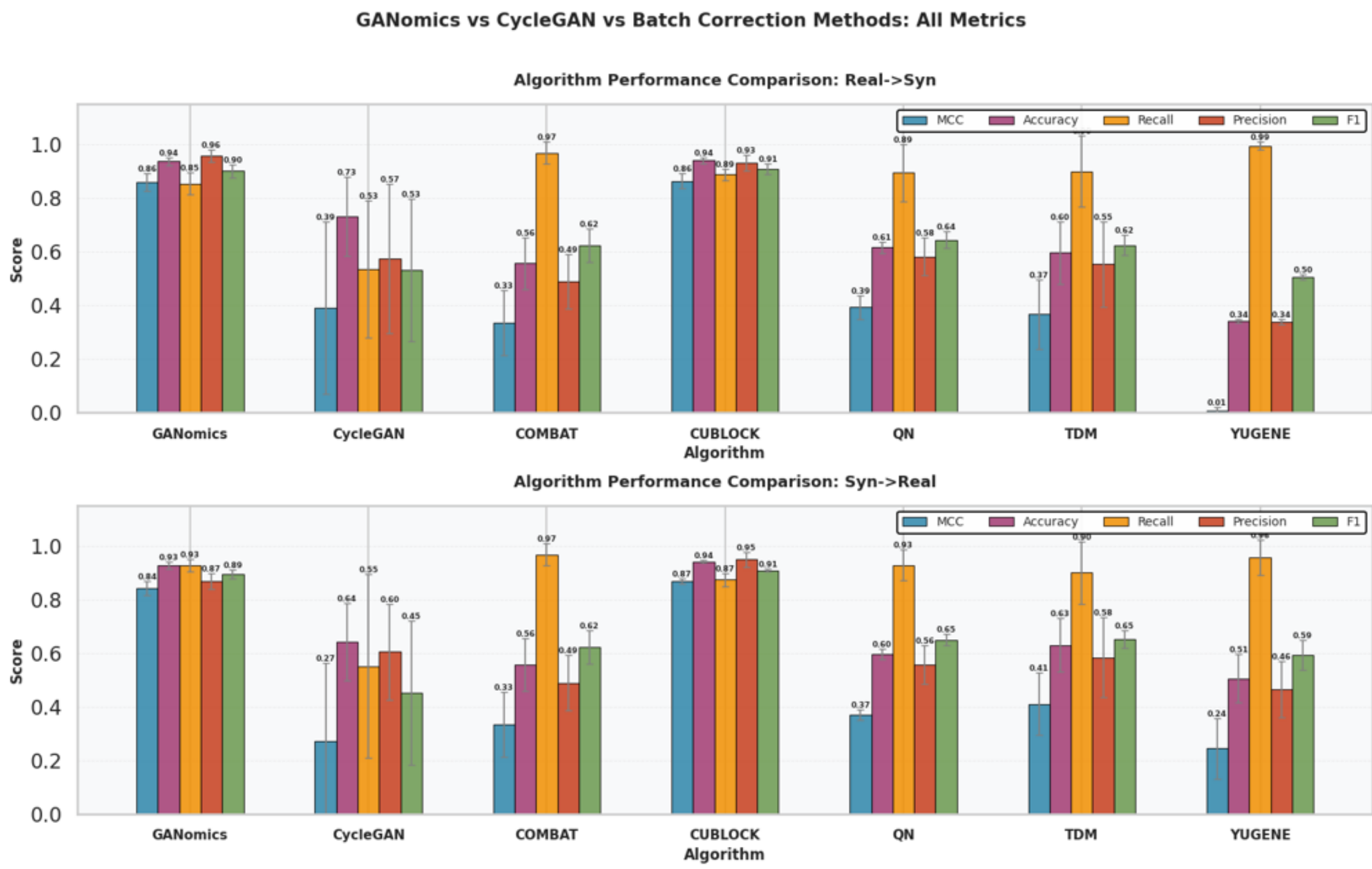

Figure S3. *Impact of feedback loss weight (β) on downstream predictive performance. Performance of Random Forest classifiers trained and evaluated using GANomics-translated data under different feedback loss weights compared to the default GANomics-50 configuration (β = 10). Results are shown for (top) Real→Synthetic and (bottom) Synthetic→Real scenarios on the NB dataset. Evaluation metrics include MCC, accuracy, recall, precision, and F1 score (mean ± variability across runs). Models with moderate feedback weights (β ≥ 5) maintain strong predictive performance comparable to the default setting, whereas β = 0 (CycleGAN) and β = 1 show substantial degradation, particularly in MCC and F1 score. These results highlight the importance of the feedback loss in preserving sample-level biological information for downstream prediction tasks.*
